# Glycosphingolipids in human abdominal leiomyosarcoma and liposarcoma

**DOI:** 10.1016/j.jbc.2026.111327

**Published:** 2026-02-26

**Authors:** Chunsheng Jin, Anders Thornell, Marta Berndsen, Karin Säljö, Eva Jennische, Susann Teneberg

**Affiliations:** 1 Department of Medical Biochemistry and Cell Biology, Institute of Biomedicine, Sahlgrenska Academy, University of Gothenburg, Gothenburg, Sweden; 2 Department of Surgery, Institute of Clinical Sciences, Sahlgrenska Academy, University of Gothenburg, Gothenburg, Sweden; 3 Region Västra Götaland, Sahlgrenska University Hospital, Gothenburg, Sweden; 4 Department of Plastic Surgery, Institute of Clinical Sciences, Sahlgrenska Academy, University of Gothenburg, Gothenburg, Sweden

**Keywords:** cancer stem cells, glycolipid structure, ganglioside, mass spectrometry (MS), tumor marker, sarcoma

## Abstract

Retroperitoneal sarcomas (liposarcomas and leiomyosarcomas) are rare and highly malignant mesenchymal tumors with poor prognosis, since they often reach a large size before detection, due to their anatomical location. Thus, development of new tumor biomarkers for early detection of these cancers is urgently needed. In search for potential sarcoma biomarkers, this study examined the glycosphingolipid profiles of one leiomyosarcoma and one liposarcoma by mass spectrometry, enzymatic hydrolysis, and by binding of carbohydrate-recognizing ligands. Detailed mass spectrometry analyses of oligosaccharides released from the glycosphingolipids showed that the liposarcoma had a number of glycosphingolipids previously found in many human tissues (the gangliosides GM3, GD3, GM2, GD1a, sialylneolactotetra- and hexaosylceramide, globo- and lactotriaosylceramide, globo- and lactotetraosylceramide, x_2_ pentaosylceramide, H type 2 penta- and heptaosylceramide, neolactotetra- and heptaosylceramide). The leiomyosarcoma had a more complex glycosphingolipid composition, and in addition to the compounds listed above, it contained sialyl-lactotetraosylceramide, sialyl-globotetraosylceramide, globopentaosylceramide/SSEA-3, sialyl-globopentaosylceramide/SSEA-4, and disialyl-globopentaosylceramide. The expression of sialyl-globopentaosylceramide/SSEA-4 in leiomyosarcoma was confirmed by immunohistochemistry. Thus, the leiomyosarcoma had several glycosphingolipids also found in human pluripotent stem cells. These insights may have important clinical implications for the development of novel diagnostic and therapeutic strategies for sarcomas.

Abnormal glycopatterns, for example, occurrence of truncated structures, precursors, or novel glycan structures, is a hallmark of cancer and is due to cancer-specific changes in glycan biosynthesis pathways, such as the altered expression of glycosyltransferases and glycosidases. These differences in glycosylation between malignant and healthy tissues may be used as specific cancer biomarkers, and many tumor biomarkers currently used in the clinic are based on glycans, as, for example, AFP (alfa-fetoprotein) for liver cancer, CEA (carcinoembryonic antigen) for colon cancer, PSA (prostate-specific antigen) for prostate cancer, CA125 for ovarian cancer, and CA19-9/sialyl-Lewis^a^ for gastrointestinal and pancreatic cancer (1).

Sarcoma of the abdomen is a relatively rare diagnosis. The most common are the gastrointestinal stromal tumor located mainly in the abdominal cavity. In the retroperitoneal cavity, the most frequent type is liposarcoma, followed by leiomyosarcoma (2). The incidence for retroperitoneal sarcomas is approximately 0.3 per 100,000 inhabitants/year (3, 4). The recurrence rate after primary surgery is generally about 30 to 40% depending on tumor volume, subtype, and location. The treatment for recurrent disease consists primarily of surgery as the oncomedical treatment often has a poor effect (5, 6). Discovering an early recurrence of a sarcoma can improve survival rate but requires radiologic surveillance every 3 to 6 months (7, 8), which is costly for society and strenuous for the patients.

Differential expression of glycans between normal cells and cancer cells occurs at the onset or during cancer progression, and identifying such changes at early disease stages may provide tools for screening and monitoring cancer patients (9, 10, 11, 12).

Previous studies about the glycosylation of leiomyosarcomas and liposarcomas are very few and scattered. Li *et al*. investigated the nonacid glycosphingolipids of a uterine leiomyosarcoma by using glycosidase digestion, permethylation analysis, and high-pressure liquid chromatography and reported the presence of lactosylceramide, globotriaosylceramide, globotetraosylceramide, and neolactotetraosylceramide. They also observed that the content of globotriaosylceramide was increased when compared to normal uterus glycosphingolipids (13). By using immunostaining on thin-layer chromatograms, liposarcoma gangliosides have been identified as GM3, sialyl-neolactotetraosylceramide, and GD3 (14).

Staining of tissue sections of leiomyosarcoma by the 14F7 monoclonal antibody recognizing the *N*-glycolyl GM3 ganglioside (Neu5Gc-GM3) has been reported by Blanco *et al*. (15). Immunohistochemistry has also demonstrated the expression of the gangliosides GD2 and GD3 in sarcomas of different histologic types, including leiomyosarcomas and liposarcomas (16).

The *N*-linked glycans of leiomyosarcoma and liposarcoma has been investigated by on-tissue digestion matrix-assisted laser desorption/ionization mass spectrometry imaging demonstrating alterations in high-mannose type glycans during tumor progression (17, 18, 19).

Since a thorough structural characterization of leiomyosarcoma and liposarcoma glycosphingolipids with the more sensitive methods available today is lacking, we have in this study explored the glycosphingolipids of retroperitoneal liposarcoma and leiomyosarcoma to characterize possible tumor markers and druggable targets. The glycosphingolipids were characterized by mass spectrometry of oligosaccharides released from the glycosphingolipids, enzymatic digestion, and by binding of a battery of carbohydrate-recognizing ligands, and the tissue distribution of selected compounds was investigated by immunohistochemistry. The glycosphingolipid profile of the liposarcoma (the gangliosides GM3, GD3, GM2, GD1a, sialylneolactotetra- and hexaosylceramide, globo- and lactotriaosylceramide, globo- and lactotetraosylceramide, x_2_ pentaosylceramide, H type 2 penta- and heptaosylceramide, neolactotetra- and heptaosylceramide) was very similar to normal human tissues (20, 21, 22, 23, 24, 25). The leiomyosarcoma had a more complex glycosphingolipid composition, and in addition to the compounds listed above, contained sialyl-lactotetraosylceramide, sialyl-globotetraosylceramide, globopentaosylceramide/SSEA-3, sialyl-globopentaosylceramide/SSEA-4, and disialyl-globopentaosylceramide, that is, glycosphingolipids also present in human pluripotent stem cells (26, 27, 28).

## Results

### Isolation of glycosphingolipids

**Figure 1:**
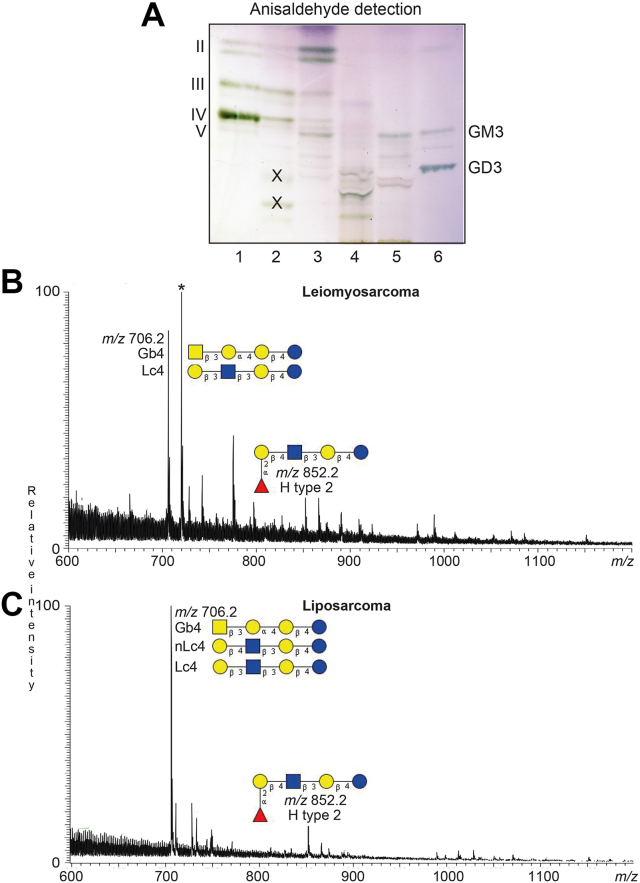
**Characterization of the total nonacid and acid glycosphingolipids from human leiomyosarcoma and liposarcoma**. *A*, thin-layer chromatography of the total nonacid and acid glycosphingolipid fractions isolated from human leiomyosarcoma and liposarcoma. The glycosphingolipids were separated on glass-backed silica gel plates, using chloroform/methanol/water 60:35:8 (by volume) as solvent system and anisaldehyde was used for detection. Lanes: Lane 1, reference total nonacid glycosphingolipids from human blood group O erythrocytes, 40 μg; lane 2, total nonacid glycosphingolipids from human leiomyosarcoma, 40 μg; lane 3, total nonacid glycosphingolipids from human liposarcoma, 40 μg; lane 4, total acid glycosphingolipids from human leiomyosarcoma, 40 μg; lane 5, total acid glycosphingolipids from human liposarcoma, 40 μg; lane 6, reference GM3 (Neu5Acα3Galβ4Glcβ1Cer) and GD3 (Neu5Acα8Neu5Acα3Galβ4Glcβ1Cer) gangliosides, 2 and 4 μg. The Roman numbers to the *left* of the chromatogram indicate the approximate number of carbohydrate units in the nonacid fractions in lanes 1 to 3. The designations GM3 and GD3 to the *right* of the chromatogram show the migration level of the GM3 and GD3 gangliosides, respectively. All bands on the chromatogram in (*A*), except these marked with X, were colored *green* with the anisaldehyde reagent and thus contained carbohydrate (29). *B* and *C*, characterization of the total nonacid glycosphingolipids from human leiomyosarcoma and liposarcoma by LC-ESI/MS. LC-ESI/MS of the oligosaccharides derived from the total nonacid glycosphingolipid fractions from human leiomyosarcoma and liposarcoma by hydrolysis with endoglycoceramidase II from *Rhodococcus* spp. The identification of oligosaccharides was based on their retention times, determined molecular masses, subsequent MS^2^ sequencing, and comparison with reference MS^2^ spectra. (*B*) Molecular ion profile from LC-ESI/MS of the oligosaccharides derived from the total nonacid glycosphingolipid fraction from human leiomyosarcoma. (*C*) Molecular ion profile from LC-ESI/MS of the oligosaccharides derived from the total nonacid glycosphingolipid fraction from human liposarcoma. Gb4, GalNAcβ3Galα4Galβ4Glc; Lc4, Galβ3GlcNAcβ3Galβ4Glc; nLc4, Galβ4GlcNAcβ3Galβ4Glc; H5 type 2, Fucα2Galβ4GlcNAcβ3Galβ4Glc. The peak marked with ∗ in (*B*) is a nonoligosaccharide contaminant.

Total acid and nonacid glycosphingolipid fractions were isolated from human leiomyosarcoma and liposarcoma by standard procedures (29). The total nonacid fraction from the leiomyosarcoma had major compounds migrating in the tri- and tetraglycosylceramide regions (Fig. 1*A*, lane 2), while the total nonacid fraction from the liposarcoma had a number of compounds migrating as di-, tri-, tetra-, and pentaosylceramides on thin-layer plates (Fig. 1*A*, lane 3). The leiomyosarcoma total acid fraction had a number of slow-migrating compounds (Fig. 1*A*, lane 4), and the liposarcoma total acid fraction had compounds comigrating with reference GM3 ganglioside and below (Fig. 1*A*, lane 5).

### Characterization of the total nonacid glycosphingolipid fractions from human leiomyosarcoma and liposarcoma by LC-ESI/MS

The glycosphingolipids in the total nonacid glycosphingolipid fractions were characterized by mass spectrometry. The total nonacid fractions were hydrolyzed with endoglycoceramidase II from *Rhodococcus* sp., and the oligosaccharides thereby obtained were characterized by LC-ESI/MS using a graphitized carbon column. This gives a resolution of isomeric oligosaccharides, and by MS^2^, a series of C-type ions is obtained, which gives the carbohydrate sequence (30). Furthermore, the MS^2^ spectra of oligosaccharides where a Hex or HexNAc residue is substituted at C-4 position have diagnostic cross-ring fragment ions of ^0,2^A and ^2,4^A cleavage, which allow determination of linkage positions (30, 31). Thus, such fragment ions are present in the MS^2^ spectra of oligosaccharides with globo (Galα4Gal) or type 2 (Galβ4GlcNAc) core chains, but not in the MS^2^ spectra obtained from oligosaccharides with isoglobo (Galα3Gal) or type 1 (Galβ3GlcNAc) core chains. Comparisons of retention times and MS^2^ spectra of oligosaccharides from reference glycosphingolipids are also used for the identification of oligosaccharides.

The first LC-ESI/MS analyses of the oligosaccharides derived from the total nonacid glycosphingolipid fractions from the human leiomyosarcoma and liposarcoma gave molecular ion profiles which in both cases had predominant [M-H^+^]^−^ ions at *m/z* 706.2 (Fig. 1, *B* and *C*). Here MS^2^ identified globo and lacto tetrasaccharides in the case of the leiomyosarcoma (Fig. S1, *A* and *B*), and globo, lacto, and neolacto tetrasaccharides in the case of the liposarcoma (Fig. S1, *D* and *E*). Both base peak chromatograms also had a minor [M-H^+^]^−^ ion at *m/z* 852.2. MS^2^ of these ions characterized the H type 2 pentasaccharide in both the leiomyosarcoma and the liposarcoma (Fig. S1, *C* and *F*).

### Separation of the total nonacid glycosphingolipids from human leiomyosarcoma and liposarcoma

**Figure 2:**
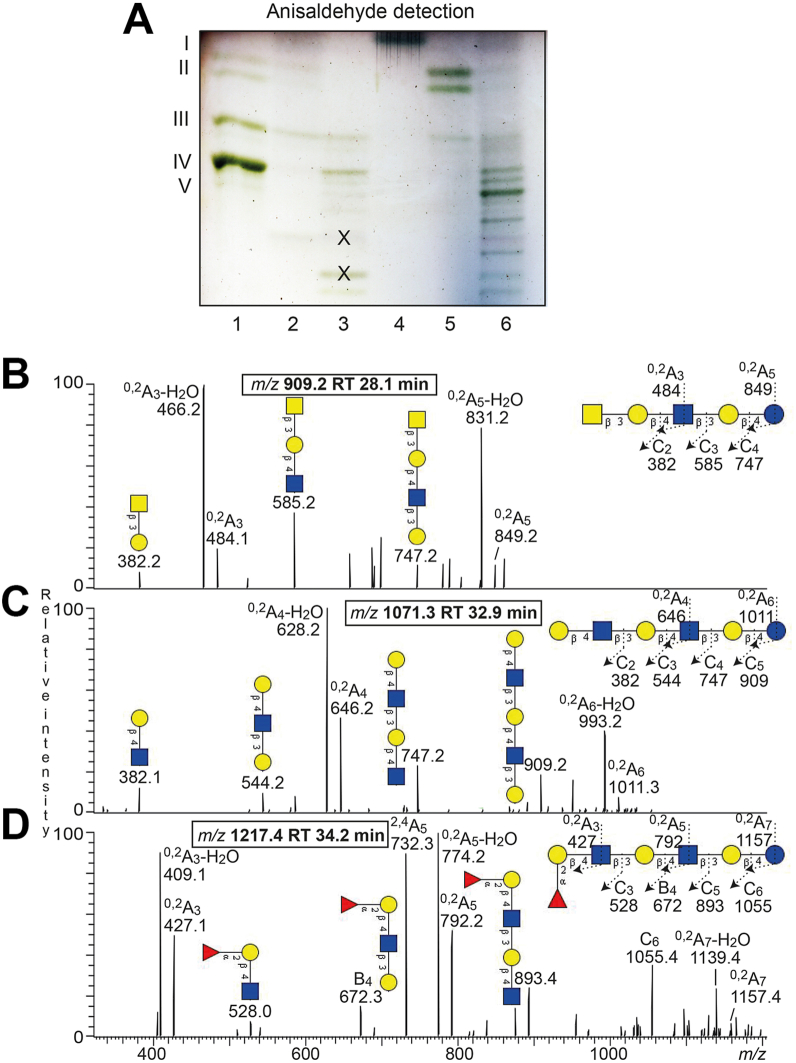
**Separation of the nonacid glycosphingolipids from human leiomyosarcoma and liposarcoma**. *A*, thin-layer chromatography of the nonacid glycosphingolipid subfractions obtained by separation of the total nonacid glycosphingolipids from human leiomyosarcoma and liposarcoma. The glycosphingolipids were chromatographed on glass-backed silica gel plates, using chloroform/methanol/water 60:35:8 (by volume) as solvent system and anisaldehyde was used for detection. Lanes: Lane 1, reference total nonacid glycosphingolipids from human blood group O erythrocytes, 40 μg; lane 2, fraction Leio-1 from human leiomyosarcoma, 4 μg; lane 3, fraction Leio-2 from human leiomyosarcoma, 4 μg; lane 4, fraction Lipo-1 from human liposarcoma, 4 μg; lane 5, fraction Lipo-2 from human liposarcoma, 4 μg; lane 6, fraction Lipo-3 from human liposarcoma, 4 μg. The Roman numbers to the *left* of the chromatogram indicate the approximate number of carbohydrate units in the bands. All bands on the chromatogram in (*A*), except these marked with X, were colored *green* with the anisaldehyde reagent and thus contained carbohydrate (29). *B*–*D*, characterization of the slow-migrating nonacid glycosphingolipids from human leiomyosarcoma (fraction Leio-2) by LC-ESI/MS. LC-ESI/MS of the oligosaccharides derived from fraction Leio-2 from human leiomyosarcoma by hydrolysis with endoglycoceramidase II from *Rhodococcus* spp. The identification of oligosaccharides was based on their retention times, determined molecular masses, subsequent MS^2^ sequencing, and comparison with reference MS^2^ spectra. *B*, MS^2^ spectrum of the [M-H^+^]^−^ ion at *m/z* 909.2 at retention time 28.1 min. *C*, MS^2^ spectrum of the [M-H^+^]^−^ ion at *m/z* 1071.3 at retention time 32.3 min. *D*, MS^2^ spectrum of the [M-H^+^]^−^ ion at *m/z* 1217.4 at retention time 33.9 min.

To enrich the slow-migrating glycosphingolipids the total non-acid glycosphingolipid fractions were next separated on Iatrobeads columns. Thereby, two glycosphingolipid-containing fractions were obtained from the leiomyosarcoma (denoted fractions Leio-1, and Leio-2). The glycosphingolipids in fraction Leio-1 migrated in the di- and triglycosylceramide regions (Fig. 2*A*, lane 2), while the main glycosphingolipids in fraction Leio-2 migrated as tri- and tetraglycosylceramides, and there were also some minor slow-migrating compounds (Fig. 2*A*, lane 3).

Separation of the nonacid glycosphingolipids from the liposarcoma resulted in three fractions denoted Lipo-1, Lipo-2, and Lipo-3. Fraction Lipo-1 contained compounds migrating as monoglycosylceramides (Fig. 2*A*, lane 4), while fraction Lipo-2 had compounds migrating as di- and triglycosylceramides (Fig. 2*A*, lane 5). Fraction Lipo-3 had glycosphingolipids migrating as tri- and tetraglycosylceramides and a serial of slow-migrating glycosphingolipids (Fig. 2*A*, lane 6).

### Characterization of the nonacid glycosphingolipid subfractions by LC-ESI/MS

Fractions Leio-1, Leio-2, Lipo-2, and Lipo-3 were subjected to endoglycoceramidase II digestion, and the oligosaccharides thereby obtained were analyzed by LC-ESI/MS, as above.

#### Fraction Leio-1

The base peak chromatogram from LC-ESI/MS of the oligosaccharides obtained from fraction Leio-1 had two major [M-H^+^]^−^ ions at *m/z* 503.1 and *m/z* 544.2. MS^2^ of these ions identified a globo (Galα4Galβ4Glc; *m/z* 503.1) and a lacto (GlcNAcβ3Galβ4Glc; *m/z* 544.2) trisaccharide, respectively (Fig. S2, *A* and *B*).

#### Fraction Leio-2

LC-ESI/MS of the oligosaccharides obtained from fraction Leio-2 gave a base peak chromatogram with seven [M-H^+^]^−^ ions. The major [M-H^+^]^−^ ions were found at *m/z* 706.2 (two peaks) and at *m/z* 852.2, and MS^2^ of these ions identified globo and lacto tetrasaccharides, and the H type 2 pentasaccharide, as above (Fig. S2, *C*–*F*).

The base peak chromatogram also had minor [M-H^+^]^−^ ions at *m/z* 868.2, *m/z* 909.2, *m/z* 1071.3, and *m/z* 1217.4. MS^2^ of the [M-H^+^]^−^ ion at *m/z* 909.2 (Fig. 2*B*) gave a series of C-type fragment ions (C_2_ at *m/z* 382.2, C_3_ at *m/z* 585.2, and C_4_ at *m/z* 747.2), demonstrating a HexNAc-Hex-HexNAc-Hex-Hex sequence. A type 2 core chain was identified by the ^0,2^A_3_-H_2_O/^0,2^A_3_ ion at *m/z* 466.2/484.1. The spectrum was very similar to the MS^2^ spectrum of the x_2_ pentasaccharide (32), and thus an x_2_ pentasaccharide (GalNAcβ3Galβ4GlcNAcβ3Galβ4Glc) was identified.

A neolacto hexasaccharide (Galβ4GlcNAcβ3Galβ4GlcNAcβ3Galβ4Glc) was characterized by MS^2^ of the [M-H^+^]^−^ ion at *m/z* 1071.3 (Fig. 2*C*). This was deduced from the C-type fragment ion series (C_2_ at *m/z* 382.1, C_3_ at *m/z* 544.2, C_4_ at *m/z* 747.2, and C_5_ at *m/z* 909.2), demonstrating a Hex-HexNAc-Hex-HexNAc-Hex-Hex carbohydrate sequence, along with the ^0,2^A_4_-H_2_O/^0,2^A_4_ fragment ion at *m/z* 628.2/646.2, which demonstrated 4-substitution of the innermost HexNAc.

A [M-H^+^]^−^ ion at *m/z* 1217.4 corresponds to a heptasaccharide with one Fuc, two HexNAc, and four Hex. The MS^2^ spectrum obtained of *m/z* 1217.4 (Fig. 2*D*) had a number of B- and C-type fragment ions (C_3_ at *m/z* 528.0, B_4_ at *m/z* 672.3, C_5_ at *m/z* 893.4, and C_6_ at *m/z* 1055.4) in line with a Fuc-Hex-HexNAc-Hex-HexNAc-Hex-Hex sequence. The ^0,2^A_3_-H_2_O/^0,2^A_3_ ion at *m/z* 409/427 demonstrated a Fuc-Hex-HexNAc terminal with 4-substitution of the HexNAc, while 4-substitution of the innermost HexNAc was demonstrated by the ^0,2^A_5_-H_2_O/^0,2^A_5_ ion at *m/z* 774.2/792.2 and the ^2,4^A_5_ ion at *m/z* 732.3. Taken together, these spectral features gave identification of a H type 2 heptasaccharide (Fucα4Galβ4GlcNAcβ3Galβ4GlcNAcβ3Galβ4Glc).

**Figure 3:**
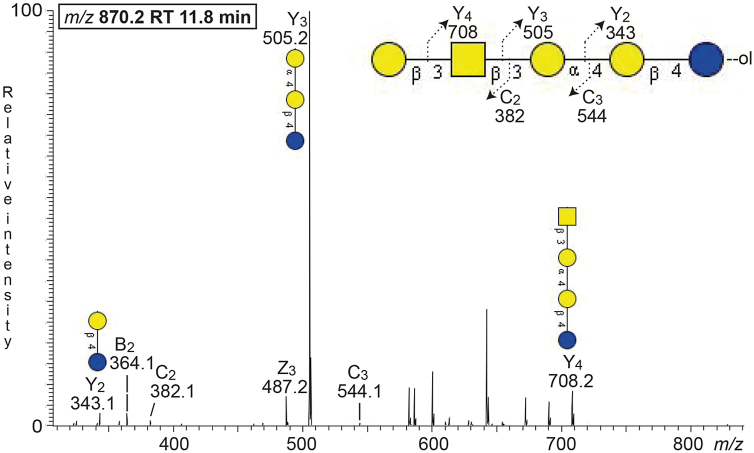
**Characterization of the slow-migrating nonacid glycosphingolipids from human leiomyosarcoma (fraction Leio-2) by LC-ESI/MS**. LC-ESI/MS of the reduced oligosaccharides derived from fraction Leio-2 from human leiomyosarcoma by hydrolysis with endoglycoceramidase II from *Rhodococcus* spp. The identification of oligosaccharides was based on their retention times, determined molecular masses, subsequent MS^2^ sequencing, and comparison with reference MS^2^ spectra. MS^2^ of the ion at *m/z* 870.2 at retention time 11.8 min.

The MS^2^ spectrum of the [M-H^+^]^−^ ion at *m/z* 868.2 did not allow a reliable determination of the carbohydrate sequence. Therefore the sample was reduced and re-analyzed by LC-ESI/MS. The spectrum obtained by MS^2^ of the ion at *m/z* 870.2 (reduced form of *m/z* 868.2) (Fig. 3) had a number of Y ions (Y_2_ at *m/z* 343.1, Y_3_ at *m/z* 505.2, Y_4_ at *m/z* 708.2), which along with the series of B and C ions (B_2_ at *m/z* 364.1, C_2_ at *m/z* 382.1, and C_3_ at *m/z* 544.1) identified a Hex-HexNAc-Hex-Hex-Hex sequence, like the globo/SSEA-3 pentasaccharide (Galβ3GalNAcβ3Galα4Galβ4Glc).

#### Fraction Lipo-2

LC-ESI/MS and MS^2^ of the oligosaccharides obtained from fraction Lipo-2 identified three oligosaccharides; globo (*m/z* 503.1) and lacto (*m/z* 544.2) trisaccharides (Fig. S3, *A* and *B*), and globo tetrasaccharide (*m/z* 706.2; data not shown).

#### Fraction Lipo-3

The oligosaccharides from fraction Lipo-3 characterized by LC-ESI/MS and MS^2^ were globo, lacto, and neolacto tetrasaccharides (*m/z* 706.2), H type 2 pentasaccharide (*m/z* 852.2), x_2_ pentasaccharide (*m/z* 909.3), and neolacto hexasaccharide (*m/z* 1071.4) (Fig. S3, *C*–*G*).

The nonacid glycosphingolipids characterized in the human leiomyosarcoma and liposarcoma by LC-ESI/MS of glycosphingolipid-derived oligosaccharides are summarized in Table 1.

**Table 1:** Nonacid glycosphingolipids identified in human leiomyosarcoma and liposarcoma^a^

*mm/z* [M-H]^-^	Trivial name	Structures with ceramides	Leiomyo-sarcoma	Lipo-sarcoma
	*Globo series*			
503	Globotri (Gb3)	Galα4Galβ4Glcβ1Cer	+	+
706	Globotetra (Gb4)	GalNAcβ3Galα4Galβ4Glcβ1Cer	+	+
868	Globopenta (Gb5) SSEA-3	Galβ3GalNAcβ3Galα4Galβ4Glcβ1Cer	+	-
	*Lact/Neolacto series*			
544	Lactotri (Lc3)	GlcNAcβ3Galβ4Glcβ1Cer	+	+
706	Lactotetra (Lc4)	Galβ3GlcNAcβ3Galβ4Glcβ1Cer	+	+
706	Neolactotetra (nLc4)	Galβ4GlcNAcβ3Galβ4Glcβ1Cer	-	+
852	H type 2 penta (H5-2)	Fucα2Galβ4GlcNAcβ3Galβ4Glcβ1Cer	+	+
909	x_2_ penta (x_2_)	GalNAcβ3Galβ4GlcNAcβ3Galβ4Glcβ1Cer	+	+
1071	Neolactohexa (nLc6)	Galβ4GlcNAcβ3Galβ4GlcNAcβ3Galβ4Glcβ1Cer	+	+
1217	H type 2 hepta (H7-2)	Fucα2Galβ4GlcNAcβ3Galβ4GlcNAcβ3Galβ4Glcβ1Cer	+	-

a The glycosphingolipids were identified by LC-ESI/MS of glycosphingolipid-derived oligosaccharides.

### Characterization of the total acid glycosphingolipid fractions from human leiomyosarcoma and liposarcoma by LC-ESI/MS

**Figure 4:**
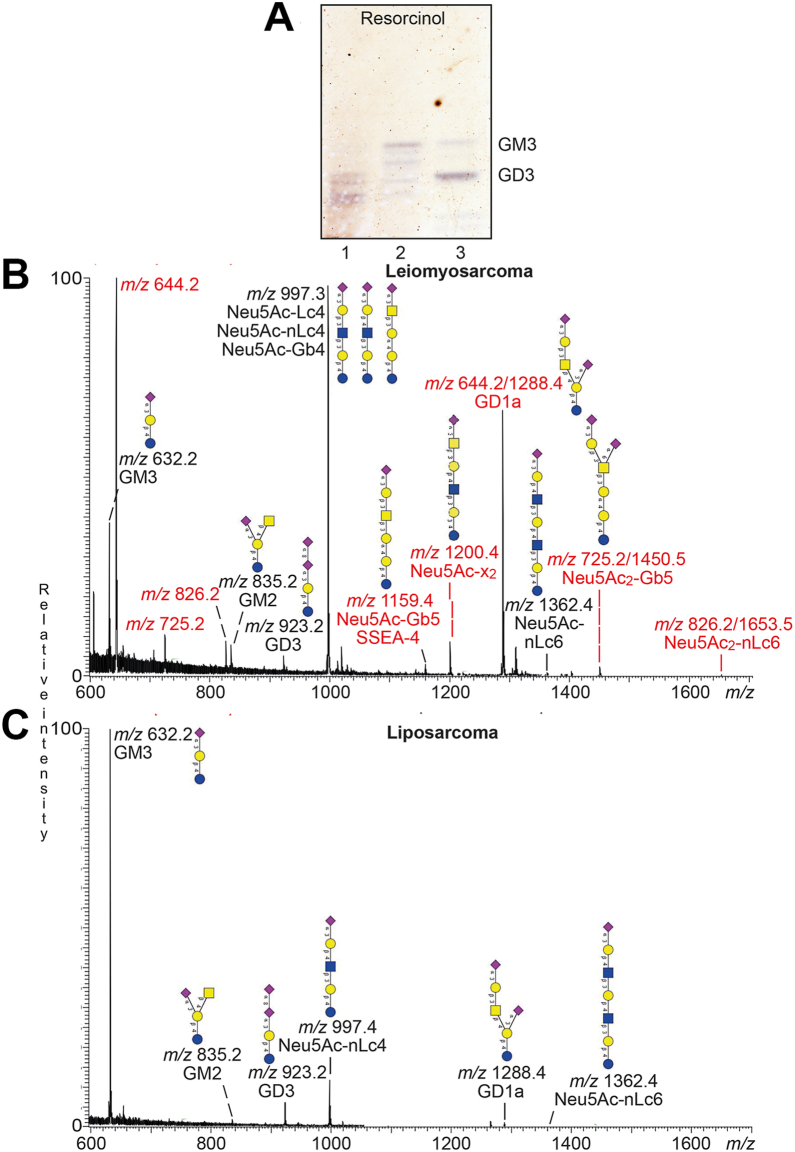
**Characterization of the acid glycosphingolipids from human leiomyosarcoma and liposarcoma by LC-ESI/MS**. *A*, thin-layer chromatography of the total acid glycosphingolipid fractions from human leiomyosarcoma and liposarcoma. The glycosphingolipids were separated on glass-backed silica gel plates, using chloroform/methanol/water 60:35:8 (by volume) as solvent system and resorcinol was used for detection. Lanes: Lane 1, total acid glycosphingolipids from human leiomyosarcoma, 40 μg; lane 2, total acid glycosphingolipids from human liposarcoma, 40 μg; lane 3, reference GM3 (Neu5Acα3Galβ4Glcβ1Cer) and GD3 (Neu5Acα8Neu5Acα3Galβ4Glcβ1Cer) gangliosides, 2 and 4 μg. *B* and *C*, LC-ESI/MS of the oligosaccharides derived from the total acid glycosphingolipid fractions from human leiomyosarcoma and liposarcoma by hydrolysis with endoglycoceramidase I. The identification of oligosaccharides was based on their retention times, determined molecular masses, subsequent MS^2^ sequencing, and comparison with reference MS^2^ spectra. (*B*) Molecular ion profile from LC-ESI/MS of the oligosaccharides derived from the total acid glycosphingolipid fraction from human leiomyosarcoma. (*C*) Molecular ion profile from LC-ESI/MS of the oligosaccharides derived from the total acid glycosphingolipid fraction from human liposarcoma. GM3, Neu5Acα3Galβ4Glc; GM2, GalNAcβ4(Neu5Acα3)Galβ4Glc; GD3, Neu5Acα8Neu5Acα3Galβ4Glc; Neu5Ac-Lc4, Neu5Acα3Galβ3GlcNAcβ3Galβ4Glc; Neu5Ac-nLc4, Neu5Acα3Galβ4GlcNAcβ3Galβ4Glc; Neu5Ac-Gb4, Neu5Acα3GalNAcβ3Galα4Galβ4Glc; Neu5Ac-Gb5/SSEA-4, Neu5Acα3Galβ3GalNAcβ3Galα4Galβ4Glc; Neu5Ac-x_2_, Neu5Acα3GalNAcβ3Galβ4GlcNAcβ3Galβ4Glc; GD1a, Neu5Acα3Galβ3GalNAcβ4(Neu5Acα3)Galβ4Glc; Neu5AcnLc6, Neu5Acα3Galβ4GlcNAcβ3Galβ4GlcNAcβ3Galβ4Glc; Neu5Ac_2_-Gb5, Neu5Acα3Galβ3(Neu5Acα6)GalNAcβ3Galα4Galβ4Glc; Neu5Ac_2_-nLc6, Neu5Acα3Galβ4GlcNAcβ3(Neu5Acα6)Galβ4GlcNAcβ3Galβ4Glc.

Figure 4*A* shows a resorcinol stained thin-layer chromatogram of the acid glycosphingolipid fractions from the leiomyosarcoma (lane 1) and liposarcoma (lane 2). The leiomyosarcoma had several gangliosides migrating at the level of the GD3 ganglioside (lane 3) and below. The main ganglioside of the liposarcoma migrated as the GM3 ganglioside, and there were also some more slow-migrating compounds.

Endoglycoceraminidase I was used for hydrolysis of the acid glycosphingolipids since this enzyme has a broader substrate specificity than endoglycoceraminidase II and allows an efficient hydrolysis of gangliosides (33). The molecular ion profiles from LC-ESI/MS of the oligosaccharides released from the acid glycosphingolipid fractions from the leiomyosarcoma and liposarcoma are shown in Figure 4, *B* and *C*. All molecular ions were subjected to MS^2^ (exemplified in Fig. S4) and the compounds identified are given in the figures. Thus, the predominant acid oligosaccharide derived from the liposarcoma was the GM3 trisaccharide (*m/z* 632.2) (Fig. 4*C*). The GM2 and GD3 tetrasaccharides (*m/z* 835.2 and *m/z* 923.2), Neu5Acα3/α6-neolacto pentasaccharide (*m/z* 997.4), GD1a hexasaccharide (*m/z* 1288.4), and Neu5Ac-neolacto heptasaccharide (*m/z* 1362.4) were also characterized.

The GM3 trisaccharide (*m/z* 632.2), the GM2 (*m/z* 835.2) and GD3 (*m/z* 923.2) tetrasaccharides, the GD1a hexasaccharide (*m/z* 644.2/1288.4), and Neu5Ac-neolacto heptasaccharide (*m/z* 1362.4) were also characterized in the leiomyosarcoma (Fig. 4*B* and Fig. S4). Here the molecular ion profile had a number of additional singly ([M-H^+^]^−^) and doubly (M-2H^+^]^2-^) charged molecular ions at *m/z* 997.3, *m/z* 1159.4, *m/z* 1200.4, *m/z* 725.2/1450.5, and *m/z* 826.2/1653.5, and the characterization of the corresponding oligosaccharides is described in the following.

#### Neu5Ac-Gb5/SSEA-4 (m/z 1159.4) and Neu5Ac_2_-Gb5 (m/z 725.2/1450.5)

**Figure 5:**
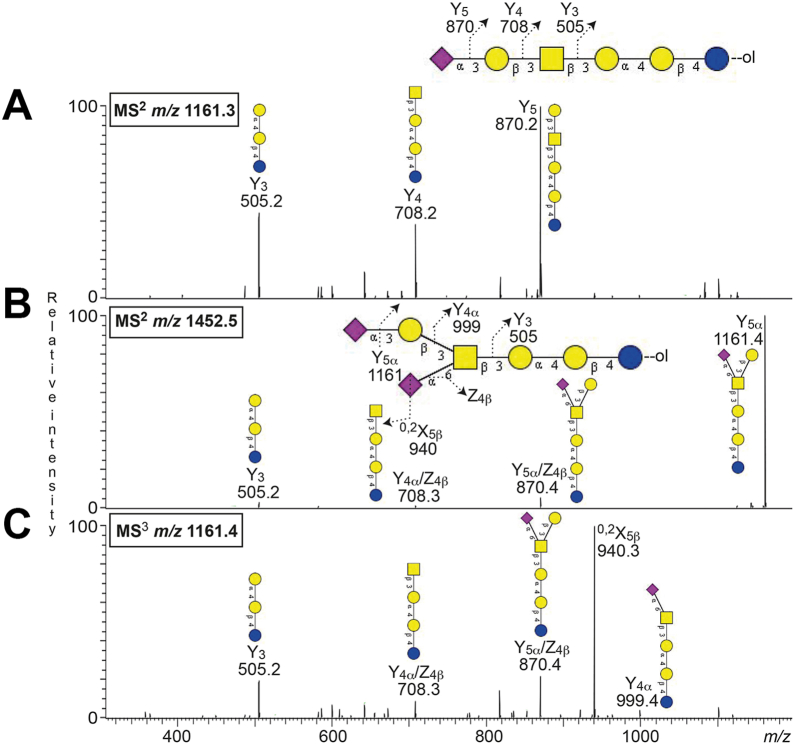
**Characterization of complex acid glycosphingolipids from human leiomyosarcoma by LC-ESI/MS**. LC-ESI/MS of the reduced oligosaccharides derived from the acid fraction from human leiomyosarcoma by hydrolysis with endoglycoceramidase I. The identification of oligosaccharides was based on their retention times, determined molecular masses, subsequent MS^2^ sequencing, and comparison with reference MS^2^ spectra. *A*, MS^2^ spectrum of the [M-H^+^]^−^ ion at *m/z* 1161.3 at retention time 13.5 min. *B*, MS^2^ spectrum of the [M-H^+^]^−^ ion at *m/z* 1452.5 at retention time 14.8 min. *C*, MS^3^ spectrum of the fragment ion at *m/z* 1161.4 obtained from the precursor ion at *m/z* 1452.5 (MS^3^*m/z* 1452.5→1161.4).

The [M-H^+^]^−^ ion at *m/z* 1159.4 corresponds to an oligosaccharide one Neu5Ac, one HexNAc, and four Hex. Here, the MS^2^ of the native oligosaccharide gave a weak spectrum that did not allow a reliable interpretation of the carbohydrate sequence. Therefore the sample was reduced and re-analyzed by LC-ESI/MS. The spectrum obtained by MS^2^ of the ion at *m/z* 1161.3 (reduced form of *m/z* 1159.4) (Fig. 5*A*) had a series of Y ions (Y_3_ at *m/z* 505.2, Y_4_ at *m/z* 708.2, and Y_5_ at *m/z* 870.2), which identified a Neu5Ac-Hex-HexNAc-Hex-Hex-Hex sequence, as the sialyl-globopenta/SSEA-4 oligosaccharide (Neu5Acα3Galβ3GalNAcβ3Galα4Galβ4Glc).

The [M-H^+^]^−^ ion at *m/z* 1450.5 indicates an oligosaccharide with two Neu5Ac, one HexNAc, and four Hex residues. MS^2^ of the ion at *m/z* 1452.5 (reduced form of *m/z* 1450.5) (Fig. 5*B*), and subsequent MS^3^ of the fragment ion at *m/z* 1161.4 (Fig. 5*C*), yielded a series of Y ions (Y_3_ at *m/z* 505.2, Y_4α_/Z_4β_ at *m/z* 708.3, Y_5α_/Z_4β_ at *m/z* 870.4, Y_4α_ at *m/z* 999.4, and Y_5α_ (or Y_4β_) at *m/z* 1161.4), consistent with a Neu5Ac-Hex-(Neu5Ac)HexNAc-Hex-Hex-Hex sequence. The spectrum had a high similarity to the spectrum in (Fig. 5*A*). There was no B_2_ ion at *m/z* 581.2, which is prominent in MS^2^ spectra of oligosaccharides with Neu5Ac-Neu5Ac sequence, as for example, the GD3 ganglioside. Instead the Y ion series indicated that one of the Neu5Ac residues was attached to the HexNAc, while the second Neu5Ac was linked to the terminal Hex. Further structural evidence was provided by MS^3^ of *m/z* 1161.4, which produced a prominent ion at *m/z* 940.3 (Fig. 5*C*). This fragment ion arises from the cross-ring cleavage of Neu5Ac which generates the [M-221] (^0,2^X_5β_), diagnostic of a Neu5Ac linked α2-6 to an internal HexNAc (34). Taken together, this tentatively identified a di-sialyl-globopenta oligosaccharide (Neu5Acα3Galβ3(Neu5Acα6)GalNAcβ3Galα4Galβ4Glc).

#### Neu5Ac-x_2_ (m/z 1200.4) and NeuAc_2_-nLc6 (m/z 826.2/1653.5)

**Figure 6:**
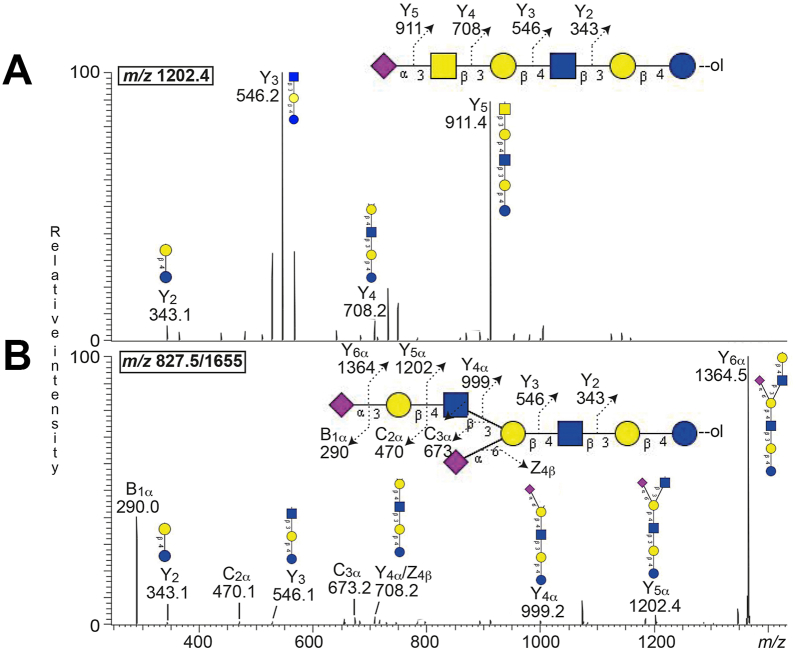
**Characterization of complex acid glycosphingolipids from human leiomyosarcoma by LC-ESI/MS**. LC-ESI/MS of the reduced oligosaccharides derived from the acid fraction from human leiomyosarcoma by hydrolysis with endoglycoceramidase I. The identification of oligosaccharides was based on their retention times, determined molecular masses, subsequent MS^2^ sequencing, and comparison with reference MS^2^ spectra. *A*, MS^2^ spectrum of the ion at *m/z* 1202.4 at retention time 15.7 min. *B*, MS^2^ spectrum of the [M-2H^+^]^2-^ ion at *m/z* 827.5 (corresponding to a [M-H^+^]^−^ ion at *m/z* 1655.0) at retention time 17.2 min.

A [M-H^+^]^−^ ion at *m/z* 1200.4 indicates an oligosaccharide composed of one Neu5Ac, two HexNAc, and three Hex residues. MS^2^ of the ion at *m/z* 1202.4 (reduced form of 1200) (Fig. 6*A*) gave a series of Y ions (Y_2_ at *m/z* 343.1, Y_3_ at *m/z* 546.2, Y_4_ at *m/z* 708.2, and Y_5_ at *m/z* 911.4), which identified a Neu5Ac-HexNAc-Hex-HexNAc-Hex-Hex sequence, as the sialyl-x_2_ oligosaccharide (Neu5Acα3GalNAcβ3Galβ4GlcNAcβ3Galβ4Glc).

A ganglioside with two Neu5Ac, two HexNAc, and four Hex residues has a molecular weight of 1653 Da. The base peak chromatogram had a doubly charged [M-2H^+^]^2-^ ion at *m/z* 826.2, which corresponds to a singly charged [M-H^+^]^−^ ion at *m/z* 1652.4. MS^2^ of the ion at *m/z* 827.5 (reduced form of *m/z* 826.2) (Fig. 6*B*) gave a series of Y ions (Y_2_ at *m/z* 343.1, Y_3_ at *m/z* 546.1, Y_4α_ at *m/z* 999.2, Y_5α_ at *m/z* 1202.4, and Y_6α_ at *m/z* 1364.5), which demonstrated a Neu5Ac_2_-Hex-HexNAc-Hex-HexNAc-Hex-Hex oligosaccharide. Once more, there was no B_2_ ion at *m/z* 581.2, ruling out a Neu5Ac-Neu5Ac sequence. The presence of the Y_4α_ ion at *m/z* 999.2 together with the Y_4α_/Z_4β_ ion at *m/z* 708.2 indicated that one of the Neu5Ac residues was attached to the internal Hex, whereas the second Neu5Ac residue was bound to the terminal Hex. This gave a tentative identification of a di-sialyl-neolactohexa oligosaccharide (Neu5Acα3Galβ4GlcNAcβ3(Neu5Acα6)Galβ4GlcNAcβ3Galβ4Glc).

#### Sialyl-neolactotetra and sialyl-globotetra (m/z 997)

There was also a broad [M-H^+^]^−^ ion at *m/z* 997.3 eluting between 29.0 min and 32.2 min in the base peak chromatogram from LC-ESI/MS of the oligosaccharides from the leiomyosarcoma acid glycosphingolipid fraction. This molecular ion corresponds to an oligosaccharide with one Neu5Ac, one HexNAc, and three Hex residues. Here MS^2^ of the [M-H^+^]^−^ ion at *m/z* 997.2 eluting at 29.1 min (Fig. S5*A*) yielded fragment ions consistent with a sialyl-neolactotetra saccharide (Neu5Acα3/6Galβ4GlcNAcβ3Galβ4Glc). In contrast, the MS^2^ spectrum obtained of the [M-H^+^]^−^ ion at *m/z* 997.2 eluting at 31.8 min (Fig. S5*B*) corresponded to a sialyl-globotetra saccharide (Neu5Acα3GalNAcβ3Galα4Galβ4Glc).

#### Sialidase treatment

**Figure 7:**
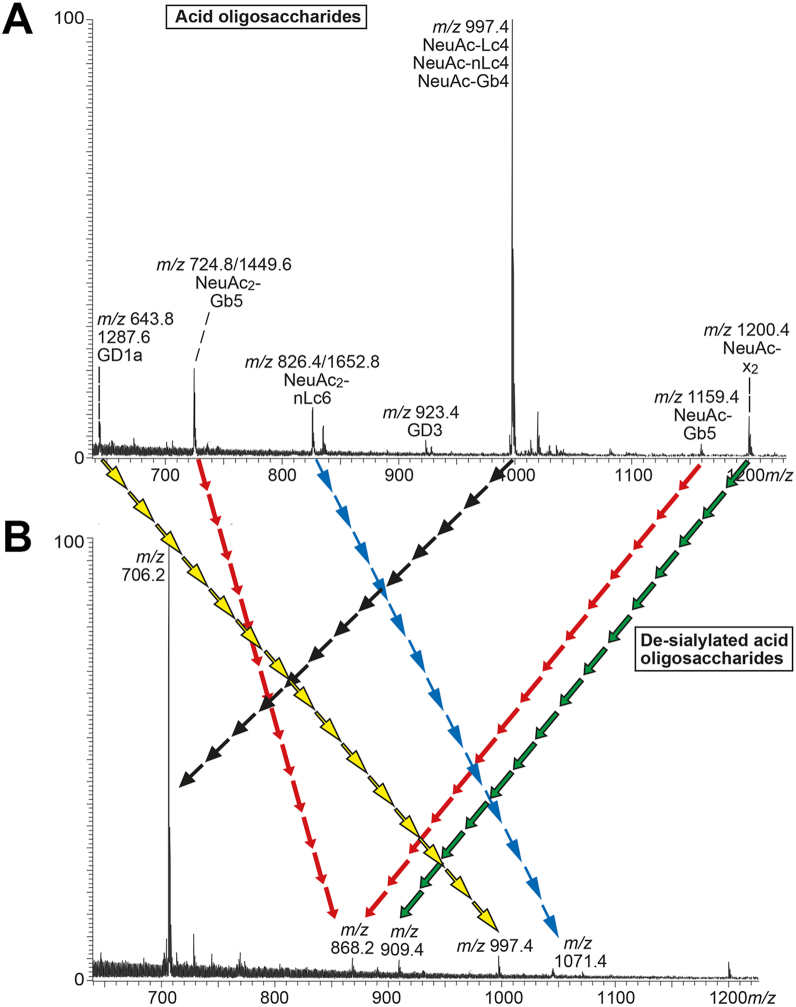
**Characterization of desialylated complex acid glycosphingolipids from human leiomyosarcoma by LC-ESI/MS**. LC-ESI/MS of the neuraminidase-treated reduced oligosaccharides derived from the acid fraction from human leiomyosarcoma by hydrolysis with endoglycoceramidase I. The identification of oligosaccharides was based on their retention times, determined molecular masses, subsequent MS^2^ sequencing, and comparison with reference MS^2^ spectra. *A*, molecular ion profile (*m/z* 600–1200) from LC-ESI/MS of the reduced oligosaccharides derived from the total acid glycosphingolipid fraction from human leiomyosarcoma. *B*, molecular ion profile (*m/z* 600–1200) from LC-ESI/MS of the de-sialylated reduced oligosaccharides derived from the total acid glycosphingolipid fraction from human leiomyosarcoma. The arrows indicate parent and related product glycans.

To substantiate the mass spectrometry data, the acid oligosaccharides from the leiomyosarcoma were digested with the α2,3/6-sialidase AmGH33 A (35), and the resulting oligosaccharides were again analyzed by LC-ESI/MS (Fig. 7*B*) and compared with the untreated oligosaccharides (Fig. 7*A*). Upon desialylation, one or two Neu5Ac (291 Da or 582 Da) were removed from the oligosaccharides and the molecular ions at *m/z* 643.8/1287.6 (identified as the GD1a hexasaccharide), *m/z* 724.8/1449.6 (di-sialo-Gb5), *m/z* 826.4/1652.8 (di-sialo-nLc6), *m/z* 997.4 (Neu5Ac-nLc4/Neu5Ac-Gb4), *m/z* 1159.4 (Neu5Ac-Gb5), and *m/z* 1200.4 (Neu5Ac-x_2_) disappeared, resulting in novel molecular ions at *m/z* 706.2 (resulting from the loss of two Neu5Ac from *m/z* 1287.6 or loss of one Neu5Ac from *m/z* 997.4), *m/z* 868.2 (loss of two Neu5Ac from *m/z* 724.8/1449.6), *m/z* 909.4 (loss of one Neu5Ac from *m/z* 1200.4), and *m/z* 1071.4 (loss of one Neu5Ac from *m/z* 826.4/1652.8).

These novel molecular ions were further analyzed by LC-ESI/MS and the base peak chromatogram thereby obtained had three [M-H^+^]^−^ ions at *m/z* 706.2 eluting at 15.9 min, 27.1 min, and 27.5 min, respectively. MS^2^ of the [M-H^+^]^−^ ion at *m/z* 706.2 eluting at 15.9 min gave a globo tetrasaccharide spectrum (Fig. S6*A*), and the MS^2^ spectrum of the [M-H^+^]^−^ ion at *m/z* 706.2 eluting at 27.1 min (Fig. S6*B*) gave identification of a lacto tetrasaccharide, while a neolacto tetrasaccharide was identified by MS^2^ of the [M-H^+^]^−^ ion at *m/z* 706.2 eluting at 27.5 min (Fig. S6*C*). The difference between lacto and neolacto tetrasaccharides is that the MS^2^ spectrum of the lacto tetrasaccharide has a C_2_/Z_3_ ion at *m/z* 202, while the MS^2^ spectrum of the neolacto tetrasaccharide contains a cross-ring cleavage at *m/z* 263/281 (^0,2^A_2_-H_2_O/^0,2^A_2_). Thus, the acid oligosaccharides derived from leiomyosarcoma contained Neu5Acα3-globo, Neu5Acα3-lacto, and Neu5Acα3/α6-neolacto pentasaccharides.

It should here be mentioned that after the treatment with sialidase, there was still a minor [M-H^+^]^−^ ion at *m/z* 997 in the base chromatogram. MS^2^ of this ion gave a weak spectrum that did not allow sequence interpretation. However, most likely, this ion represents the GM1 ganglioside (Galβ3GalNAβ4(Neu5Acα3)Galβ4Glcβ1Cer) derived from the GD1a ganglioside (Neu5Acα3Galβ3GalNAβ4(Neu5Acα3)Galβ4Glcβ1Cer) by removal of the terminal α3-linked Neu5Ac, since the Neu5Ac linked to the internal galactose of GM1 is resistant to sialidase treatment (36).

MS^2^ of the [M-H^+^]^−^ ion at *m/z* 868.2 in the sialidase-treated sample (Fig. S6*D*) gave a spectrum with high similarity to the MS^2^ spectrum of the globo/SSEA-3 pentasaccharide in Figure 3. The MS^2^ spectrum of the [M-H^+^]^−^ ion at *m/z* 909.3 in the sialidase-treated sample (Fig. S6*E*) resembled the MS^2^ spectrum of the x_2_ pentasaccharide (Fig. 2*B*), and the MS^2^ spectrum of the [M-H^+^]^−^ ion at *m/z* 1071.4 (Fig. S6*F*) was very similar to the MS^2^ spectrum of the neolacto hexasaccharide (Fig. 2*C*).

Thus, LC-ESI/MS of the desialylated sample confirmed the presence of Neu5Acα3-globotetra, Neu5Acα3-lactotetra and Neu5Acα3/α6-neolactotetra, Neu5Ac-globopenta/Neu5Ac_2_-globopenta, Neu5Ac-x_2_ penta, and Neu5Ac-neolactohexa/Neu5Ac_2_-neolactohexa saccharides in the acid oligosaccharide sample from leiomyosarcoma. The acid glycosphingolipids characterized in human leiomyosarcoma and liposarcoma by LC-ESI/MS of glycosphingolipid-derived oligosaccharides are summarized in Table 2.

**Table 2:** Acid glycosphingolipids identified in human leiomyosarcoma and liposarcoma^a^

*m/z* [M-H]^-^/[M-2H]^2-^	Trivial name	Structures with ceramides	Leiomyo-sarcoma	Lipo-sarcoma
632	GM3	Neu5Acα3Galβ4Glcβ1Cer	+	+
835	GM2	GalNAcβ4 (Neu5Acα3)Galβ4Glcβ1Cer	+	+
923	GD3	Neu5Acα8Neu5Acα3Galβ4Glcβ1Cer	+	+
997	**Sialyl-lactotetra**^b^	**Neu5Acα3Galβ3GlcNAcβ3Galβ4Glcβ1Cer**	**+**	**-**
997	Sialyl-neolactotetra	Neu5Acα3/α6Galβ4GlcNAcβ3Galβ4Glcβ1Cer	+	+
997	**Sialyl-globotetra**	**Neu5Acα3GalNAcβ3Galα4Galβ4Glcβ1Cer**	**+**	**-**
**1159**	**Sialyl-globopenta/SSEA-4**	**NeuAcα3Galβ3GalNAcβ3Galα4Galβ4Glcβ1Cer**	**+**	**-**
**1200**	**Sialyl-x_2_**	**Neu5Acα3GalNAcβ3Galβ4GlcNAcβ3Galβ4Glcβ1Cer**	+	-
644/1288	GD1a	Neu5Acα3Galβ3GalNAcβ4 (Neu5Acα3)Galβ4Glcβ1Cer	+	+
1362	Sialyl-neolactohexa	Neu5Acα3Galβ4GlcNAcβ3Galβ4GlcNAcβ3Galβ4Glcβ1Cer	+	+
**725/1450**	**Disialyl-globopenta**	**NeuAcα3Galβ3 (NeuAcα6)GalNAcβ3Galα4Galβ4Glcβ1Cer**	**+**	**-**
**826/1653**	**Disialyl-neolactohexa**	**Neu5Acα3Galβ4GlcNAcβ3 (Neu5Acα6)Galβ4GlcNAcβ3Galβ4Glcβ1Cer**	**+**	**-**

a The glycosphingolipids were identified by LC-ESI/MS of glycosphingolipid derived oligosaccharides obtained from the total acid glycosphingolipid fractions isolated from human leiomyosarcoma and liposarcoma.

b The glycosphingolipids present only in the leiomyosarcoma are in bold.

The relative percentage abundance from peak area measurements of the various reduced oligosaccharides from the total nonacid and acid glycosphingolipid fractions from leiomyosarcoma and liposarcoma are summarized in Table 3. This shows that the GM3 ganglioside was a major ganglioside of the liposarcoma. Other major components were lactotetraosylceramide, neolactotetraosylceramide, and sialyl-neolactotetraosylceramide.

**Table 3:** Relative percentage abundance of the glycosphingolipid-derived oligosaccharides from the total nonacid and acid fractions from leiomyosarcoma and liposarcoma^a^

Mass	Composition	Putative structure	Trivial name	Leio^b^ non-acid	Intensity	%	Lipo^c^ non-acid	Intensity	%
				RT (min)			RT (min)		
505	Hex3	Galα4Galβ4Glc-ol	Gb3	11.58	16,587.7	9.51	10.6	70,198.6	3.66
546	Hex2HexNAc1	GlcNAcβ3Galβ4Glc-ol	Lc3	18.75	13,227.1	7.58	13.75	95,595.6	4.98
708-1	Hex3HexNAc1	GalNAcβ3Galα4Galβ4Glc-ol	Gb4	14.16	10,810.2	6.20	11.9	40,211.5	2.10
708-2	Hex3HexNAc1	Galβ3GlcNAcβ3Galβ4Glc-ol	Lc4	25.03	106,081.8	60.81	17.39	684,782.0	35.69
708-3	Hex3HexNAc1	Galβ4GlcNAcβ3Galβ4Glc-ol	nLc4	nd^d^	0	0.0	17.7	709,038.7	36.95
854	Fuc1Hex3HexNAc1	Fucα2Galβ4GlcNAcβ3Galβ4Glc-ol	H5-2	28.23	17,365.1	9.95	19.21	166,024.6	8.65
911-1	Hex3HexNAc2	HexNAcHexNAcHexHexHex-ol		18.93	2284.5	1.31	nd	0	0.0
911-2	Hex3HexNAc2	GalNAcβ3Galβ4GlcNAcβ3Galβ4Glc-ol	x_2_-5	nd	0	0.0	20.01	13,909.9	0.72
1073	Hex4HexNAc2	Galβ4GlcNAcβ3Galβ4GlcNAcβ3Galβ4Glc-ol	nLc6	34.28	3736.84	2.14	22.78	99,185.8	5.17
1219	Fuc1Hex4HexNAc2	Fucα2Galβ4GlcNAcβ3Galβ4GlcNAcβ3Galβ4Glc-ol	H7-2	35.10	4368.8	2.50	23.07	39,955.8	2.08
Sum					174,462.0			1,918,902.5	

Mass	Composition	Putative structure	Trivial name	Leio^b^ acid	Intensity	%	Lipo^c^ acid	Intensity	%
				RT (min)			RT (min)		
634	Neu5Ac1Hex2	Neu5Acα3Galβ4Glc-ol	GM3	15.65	216,104.3	8.15	15.59	846,139	70.17
835	Neu5Ac1Hex2HexNAc1	GalNAcβ4 (Neu5Acα3)Galβ4Glc-ol	GM2	10.01	1099.6	0.04	9.95	11,678.8	0.097
925	Neu5Ac2Hex2	Neu5Acα8Neu5Acα3Galβ4Glc-ol	GD3	13.49	18,842.8	0.71	13.49	50,763	4.21
999-1	Neu5Ac1Hex3HexNAc1	Neu5Acα3Galβ3GlcNAcβ3Galβ4Glc-ol	Sialyl-Lc4	19.33	894,431.5	33.74	nd	0	0.0
999-2	Neu5Ac1Hex3HexNAc1	Neu5Acα3Galβ4GlcNAcβ3Galβ4Glc-ol	Sialyl-nLc4	21.08	794,594.7	29.97	21.06	188,565.7	15.64
1161	Neu5Ac1Hex4HexNAc1	NeuAcα3Galβ3GalNAcβ3Galα4Galβ4Glc-ol	SSEA-4	13.42	17,591.3	0.66	nd	0	0.0
1202	Neu5Ac1Hex3HexNAc2	Neu5Acα3GalNAcβ3Galβ4GlcNAcβ3Galβ4Glc-ol	Sialyl-x_2_	16.55	2079.4	0.08	nd	0	0.0
1290	Neu5Ac2Hex3HexNAc1	Neu5Acα3Galβ3GalNAcβ4 (Neu5Acα3)Galβ4Glc-ol	GD1a	11.66	65,906.3	2.49	11.59	16,254.7	1.35
1364	Neu5Ac1Hex4HexNAc2	Neu5Acα3Galβ4GlcNAcβ3Galβ4GlcNAcβ3Galβ4Glc-ol	Sialyl-nLc6	25.41	395,709.8	14.93	25.34	92,471.7	7.67
1452	Neu5Ac2Hex4HexNAc1	NeuAcα3Galβ3 (NeuAcα6)GalNAcβ3Galα4Galβ4Glc-ol	Disialyl-Gb5	14.99	163,024.1	6.15	nd	0	0.0
1655	Neu5Ac2Hex4HexNAc2	Neu5Acα3Galβ4GlcNAcβ3 (Neu5Acα6)Galβ4GlcNAcβ3Galβ4Glcβ-ol	Disialyl-nLc6	16.87	81,720.1	3.08	nd	0	0.0
Sum					12,576.5			1,205,872.9	

a The relative percentage abundance was obtained by peak area measurements of the glycosphingolipid-derived oligosaccharides from the reduced total nonacid and acid fractions from leiomyosarcoma and liposarcoma.

b Leio, leiomyosarcoma.

c Lipo, liposarcoma.

d nd, not determined.

The predominant gangliosides of the leiomyosarcoma were sialyl-lactotetraosylceramide, sialyl-neolactotetraosylceramide, and sialyl-neolactohexaosylceramide, while lactotetraosylceramide was the major nonacid glycosphingolipid. Stem cell markers apart from Neu5Ac-lactohexaosylceramide (sialyl-globopentaosylceramide/SSEA-4 and disialyl-globopentaosylceramide) were present in low amounts.

Curiously, in the LC-ESI/MS analysis of the reduced oligosaccharides from the leiomyosarcoma, total nonacid glycosphingolipid fraction [M-H^+^]^−^ ion at *m/z* 911.4 eluting at 18.9 min was observed. Here MS^2^ gave a C_2_ fragment ion at *m/z* 423.3 and an Y_3_ ion at *m/z* 505.2, indicating a HexNAc-HexNAc-Hex-Hex-Hex pentasaccharide, indicative of a Forssmann-like structure. However, no such pentasaccharide was found by MS^2^ of the native oligosaccharides from the leiomyosarcoma total nonacid oligosaccharides or by MS^2^ of the subfraction Leio-2, where MS^2^ of *m/z* 909.2 instead demonstrated an x_2_ pentasaccharide (Fig. 2*B*). This difference likely reflects altered chromatographic or ionization behavior of the alditol forms generated by reduction, which often can enhance detection of otherwise minor or labile species. Thus, the compound may be present in both forms, but was only detectable under reducing conditions.

### Chromatogram-binding assays

**Figure 8:**
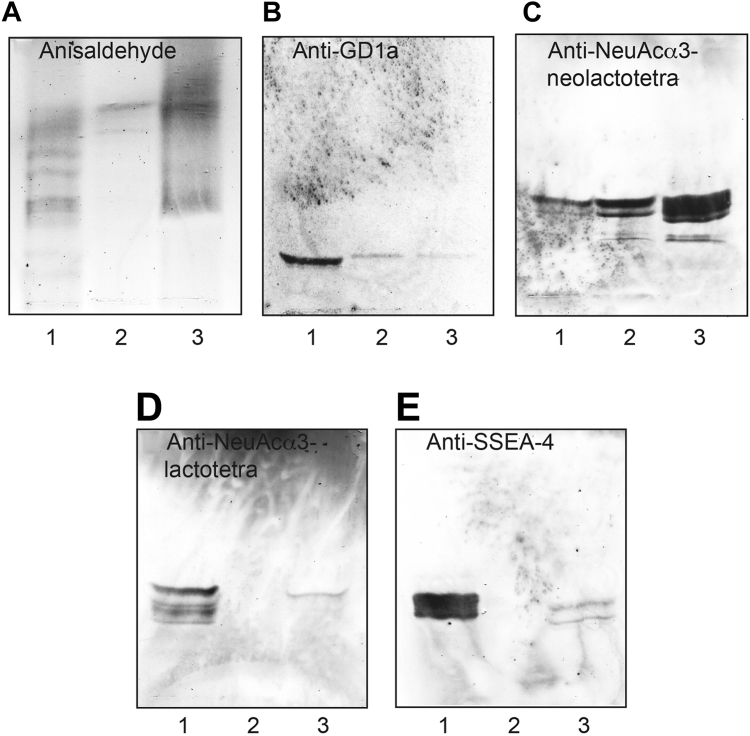
**Characterization of the acid glycosphingolipids of human leiomyosarcoma and liposarcoma by binding of monoclonal antibodies**. Thin-layer chromatogram after detection with anisaldehyde (*A*), and thin-layer chromatograms immunostained with monoclonal antibodies directed against GD1a (*B*), Neu5Acα3-neolactotetra (*C*), Neu5Acα3-lactotetra (*D*), and sialyl-globopenta/SSEA-4 (*E*). The lanes were as follows: lane 1, total acid glycosphingolipids of human leiomyosarcoma, 80 μg: lane 2, total acid glycosphingolipids of liposarcoma, 80 μg: lane 3, reference acid glycosphingolipids of human lung cancer liver metastasis, 40 μg.

To substantiate the data from mass spectrometry, the binding of a number of carbohydrate-binding antibodies to the acid glycosphingolipid fractions from human leiomyosarcoma and liposarcoma was examined (Fig. 8). The binding of anti-GD1a antibodies to the acid fractions of leiomyosarcoma and liposarcoma (Fig. 8*B*, lanes 1–2) supported the presence of the GD1a ganglioside (Neu5Acα3Galβ3GalNAcβ4(Neu5Acα3)Galβ4Glcβ1Cer), while the binding of antibodies directed against Neu5Acα3-neolacto (Fig. 8*C*, lanes 1–2) was in line with the presence of Neu5Acα3-neolactotetraosylceramide (Neu5Acα3Galβ4GlcNAcβ3Galβ4Glcβ1Cer). The antibodies directed against Neu5Acα3-lacto and SSEA-4 bound only to the leiomyosarcoma fraction (Fig. 8, *D* and *E*, lane 1) supporting the presence of Neu5Acα3-lactotetraosylceramide/sialyl-lactotetraosylceramide (Neu5Acα3Galβ3GlcNAcβ3Galβ4Glcβ1Cer) and SSEA-4/sialyl-globopentaosylceramide (Neu5Acα3Galβ3GalNAcβ3Galα4Galβ4Glcβ1Cer) in this tumor material.

### Immunohistochemistry

**Figure 9:**
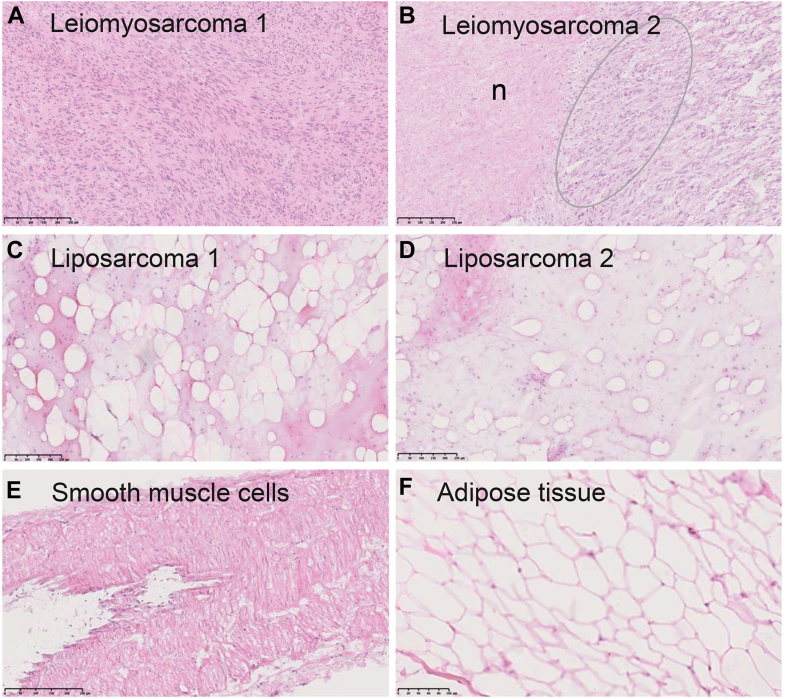
**General histology appearance of tissue samples after hematoxylin/eosin staining of cryostat sections**. *A*, leiomyosarcoma 1. *B*, leiomyosarcoma 2, n necrotic area, circle marks area with SSEA-4–positive cells. *C*, liposarcoma 1. *D*, liposarcoma 2. *E*, normal smooth muscle cells of a human muscular artery. *F*, normal human adipose tissue. Hematoxylin/eosin, bars = 250 μm.

Two Trojani grade 2 leiomyosarcomas, two Trojani grade 1 liposarcomas, normal human adipose tissue, and a normal human muscular artery were first stained by hematoxylin/eosin, showing that leiomyosarcoma 1 was homogenous with spindle shaped cells with plump, blunt ended nuclei set in long intersecting fascicles (Fig. 9*A*). Leiomyosarcoma 2 was more heterogenous and had long intersecting or haphazard fascicles, and also areas of necrosis (Fig. 9*B*). The two liposarcomas both had a mixture of adipocytes of different sizes and scattered hyperchromatic, pleomorphic, and irregular atypical cells (Fig. 9, *C* and *D*) and a large amount of extracellular matrix.

The muscular artery had smooth muscle cells circumferentially arranged in the tunica media (Fig. 9*E*), and the adipose tissue had mainly adipocytes organized into lobules and with very little extracellular matrix between cells (Fig. 9*F*).

**Figure 10:**
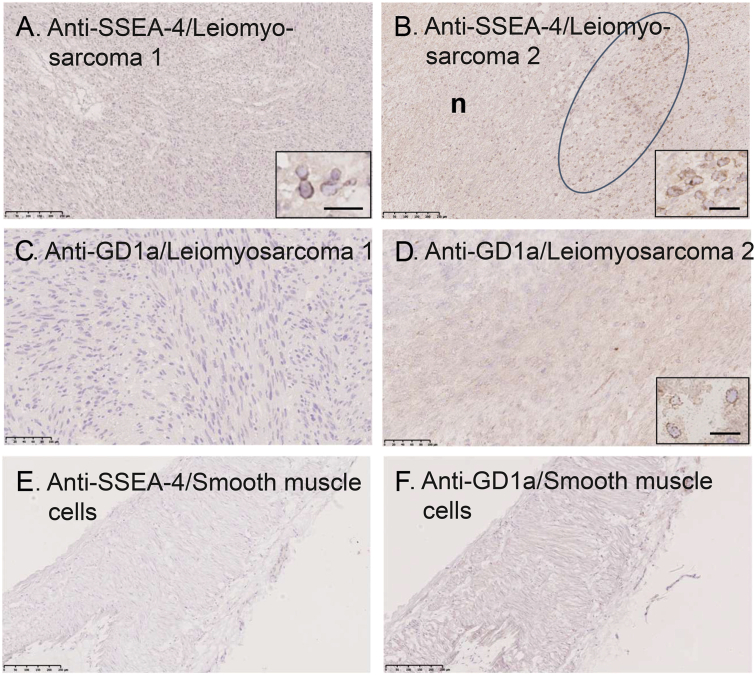
**GD1a and the sialyl-globopenta/SSEA-4 epitopes are expressed on the cell surface of leiomyosarcoma**. Immunohistochemistry of Trojani grade 2 leiomyosarcomas and normal smooth muscle cells of a muscular artery immunostained with monoclonal antibodies directed against the sialyl-globopenta/SSEA-4 epitope (*A*, *B*, and *E*) and the GD1a ganglioside (*C*, *D*, and *F*). Antibody binding was demonstrated with a peroxidase-conjugated secondary antibody/DAB giving a brown reaction product. Nuclei were counterstained with hematoxylin, *blue*. Bars represent 250 μm (*A*, *B*, *E*, *F*), 100 μm (*C* and *D*). *A*, in leiomyosarcoma 1, a few SSEA-4–postive cells could be identified; insert shows larger magnification (bar in insert 20 μm). *B*, in leiomyosarcoma 2, the circle marks an area with a large number of SSEA-4–postive cells; insert shows larger magnification (bar in insert 20 μm), n = necrotic area. *C*, in leiomyosarcoma I, no GD1a-positive cells were identified. *D*, in leiomyosarcoma 2, GD1a-positive cells were abundant; insert shows larger magnification (bar in insert 20 μm). There was no staining of the normal smooth muscle cells (SMC) of the muscular artery by the anti-sialyl-globopenta/SSEA-4 antibody (*E*) or the anti-GD1a antibody (*F*).

Next the expression of some of the carbohydrate antigens was investigated by immunohistochemistry. Leiomyosarcoma 1 had a small percentage of sialyl-globopenta/SSEA-4–positive cells (Fig. 10*A*), while in leiomyosarcoma 2, several groups of small rounded cells positive for sialyl-globopenta/SSEA-4 were found, usually in the vicinity of necrotic part of the tumor (Fig. 10*B*). The anti-GD1a antibody also gave positive staining of small rounded cells in leiomyosarcoma 2 (Fig. 10*D*), whereas leiomyosarcoma 1 was negative for anti-GD1a (Fig. 10*C*). No staining of the normal smooth muscle cells of the muscular artery was obtained with the anti-sialyl-globopenta/SSEA-4 or anti-GD1a antibodies (Fig. 10, *E* and *F*).

Investigation of the expression of sialyl-globopenta/SSEA-4 in the two liposarcomas by immunohistochemistry gave a negative outcome (Fig. S7, *A* and *B*). The anti-GD1a antibody gave a distinct cell surface staining of the adipocytes of liposarcoma 1, while the atypical cells were negative (Fig. S7*C*). Liposarcoma 2 was negative for the anti-GD1a antibody (Fig. S7*D*), and the normal adipocytes were not stained with the anti-sialyl-globopenta/SSEA-4 or anti-GD1a antibodies (Fig. S7, *E* and *F*).

There was no distinct cell binding to the leiomyosarcomas or the liposarcomas when using the anti-globopenta/SSEA-3, anti-sialyl-lactotetra, or anti-sialyl-neolactotetra antibodies.

## Discussion

Sarcomas are relatively rare, but these tumors have an aggressive biology which, together with its late detection, results in most patients having unresectable or metastatic disease at the time of diagnosis. No tumor specific biomarkers are currently available for diagnostics, prognostic evaluation, or treatment monitoring of liposarcomas or leiomyosarcomas. In this study, we have evaluated the potential of glycosphingolipids as liposarcoma and leiomyosarcoma biomarkers, by isolation and characterizing tumor glycosphingolipids.

The glycosphingolipids characterized by LC-ESI/MS of oligosaccharides released from the glycosphingolipids in the liposarcoma (globo- and lactotriaosylceramide, globo- and lactotetraosylceramide, x_2_ pentaosylceramide, H type 2 penta- and heptaosylceramide, neolactotetra- and heptaosylceramide, and the gangliosides GM3, GD3, GM2, GD1a, sialyl-neolactotetra- and hexaosylceramide) have previously been found in many normal human tissues, as for example, human erythrocytes (20), brain (21), kidney (22), stomach (23), pancreas (24), parathyroid, and thyroid glands (25).

The glycosphingolipid composition of the leiomyosarcoma was more complex, and in addition to the compounds listed above, we here provide the first evidence of the expression of sialyl-lactotetra, sialyl-globotetra, globopenta/SSEA-3, sialyl-globopenta/SSEA-4, and disialyl-globopenta in this tumor. A disialyl-neolactohexaosylceramide (Neu5Acα3Galβ4GlcNAcβ3(Neu5Acα6)Galβ4GlcNAcβ3Galβ4Glcβ1Cer) was also tentatively identified and this is to our knowledge a novel glycosphingolipid structure.

Furthermore, immunohistochemistry showed a distinct cell surface anti-sialyl-globopenta/SSEA-4 staining on an undifferentiated leiomyosarcoma sample, and the more differentiated leiomyosarcoma sample also had a small percentage of sialyl-globopenta/SSEA-4–positive cells.

Thus, all three leiomyosarcomas examined (one by glycosphingolipid characterization and two by immunohistochemistry) were SSEA-4 positive, whereas this stem cell marker was not found in the three liposarcomas or in normal human fat or normal smooth muscle cells of a muscular artery.

Interestingly, in our previous studies of glycosphingolipids in human pluripotent stem cells (human embryonic stem cells (hESC) and human-induced pluripotent stem cells (hiPSC)), these leiomyosarcoma glycosphingolipids were also characterized (27, 28, 37). Globopenta/SSEA-3 and sialyl-globopenta/SSEA-4 are established stem cell markers (38). Sialyl-lactotetra was suggested to be a novel marker of human pluripotent stem cells since there was a high cell surface expression of sialyl-lactotetra on hESC and hiPSC, while the sialyl-lactotetra epitope was rapidly downregulated upon differentiation of hESC into hepatocyte-like cells and cardiomyocyte-like cells (28), and differentiation of hiPSC into neural stem cells (37). Thus, the leiomyosarcoma had several glycosphingolipids also found in human pluripotent stem cells.

Features of stemness such as self-renewal and differentiation capacity are common characteristics of cancer cells and pluripotent stem cells. According to the cancer stem cell hypothesis, tumors have a subpopulation of cells, called cancer stem cells, which induce tumor onset and development and also contribute to chemoresistance and metastasis of cancer cells (39, 40, 41, 42, 43). Several of the markers used for cancer stem cell identification are surface markers present on human embryonic stem cells or adult stem cells, like, for example, SSEA-3 and SSEA-4 (44). Thus, the SSEA-4–positive cells found in the leiomyosarcomas are most likely cancer stem cells.

The cell surface markers used for definition and characterization of human pluripotent stem cells may also serve as targets of cancer therapy, as, for example, antibody developments, generation of CAR-T cells, and production of vaccines. Today there are several ongoing clinical trials assessing anti-globo-H and anti-SSEA-4 treatments, mainly in breast cancer (reviewed in (45)).

Noteworthily, it was recently reported that in Ewing sarcoma, the expression of the ganglioside SSEA-4 is associated with aggressive tumor growth and by engineering of T cells with an SSEA-4–specific CAR, an effective antigen-specific cytolysis of Ewing sarcoma cells was obtained (46).

Studies with structural characterization of the glycosphingolipids of the normal human equivalents of liposarcomas and leiomyosarcomas, that is, human adipose tissue or smooth muscle, are rare. Ohashi has reported that the GM3 ganglioside was the major ganglioside of human adipose tissue (47). Furthermore, using antibody binding on thin-layer chromatograms, Kubo *et al*. found that human omental fat contained the gangliosides GM3, GD1a, and GD3 along with an unidentified compound (14). In this study, the gangliosides from a human retroperitoneal liposarcoma were identified as GM3, Neu5Ac-neolactotetraosylceramide, and GD3.

By using antibody binding on thin-layer chromatograms with glycosphingolipids from cultured smooth muscle cells derived from human umbilical cord veins, Gillard *et al*. found that the major nonacid compounds were lactosylceramide, globotri- and globotetraosylceramide. Small amounts of neolactotetraosylceramide, Le^x^ pentaosylceramide, and globopentaosylceramide/SSEA-3 were also present (48). The major gangliosides were GM3 and Neu5Acα3-neolactotetraosylceramide, whereas no GD3 ganglioside was detected.

Thus, the absence of comprehensive characterization of the glycosphingolipids of control healthy tissues, that is, human adipose tissue and smooth muscle cells, is a limitation of our study.

Another major limitation of this study is the low number of tumor samples, since we only had access to three leiomyosarcomas and three liposarcomas. Since the characters of cancer cells are highly very heterogeneous between different tumors and also within tumor types, further studies on more sarcomas at different stages of tumor development are warranted.

Blanco *et al*. have reported that tissue sections of leiomyosarcoma are stained by the 14F7 monoclonal antibody recognizing the *N*-glycolyl GM3 ganglioside (15). However, no Neu5Gc-GM3 (*m/z* 648) was detected in our study.

One suggested biomarker for sarcomas is endosialin, also named TEM1 and CD248. Endosialin is a transmembrane glycoprotein which is not present in healthy adult human tissues, but expressed in the stroma and neo-vasculature of many human carcinomas and also expressed in the tumor cells of mesenchymal origin, including sarcomas (reviewed in (49)). Several endosialin-targeted antibodies with preliminary antitumor effects have recently been developed, and the development of CAR-T therapy that targets endosialin is underway (50, 51).

In general, patients with primary high-grade retroperitoneal sarcomas have a poor prognosis, and the 5-years disease-specific survival is less than 40% (5). There are currently no molecular markers for detection and monitoring treatment of these tumors, which suggests that our findings of expression of stem cell markers in leiomyosarcoma could be of clinical value. However, prospective studies are required to evaluate these findings.

## Experimental procedures

### Tissues

The study was conducted according to the tenets of the Declaration of Helsinki. The leiomyosarcomas and liposarcomas were collected at the Sahlgrenska University Hospital, with approval from the Regional Ethics Committee of Gothenburg (No. 2022-00614-01 (decision 2022-03-01)). All patients provided written informed consents before enrollment in the study. The muscular artery and the normal adipose tissue were archive materials.

### Isolation of glycosphingolipids

The tumors were kept at −70 °C. The method described by Karlsson (29) was used for isolation of total acid and total nonacid glycosphingolipids from one leiomyosarcoma and one liposarcoma. The tumors were lyophilized, followed by Soxhlet extraction with mixtures of chloroform and methanol (2:1 and 1:9, by volume). The resulting material was pooled and subjected to mild alkaline hydrolysis followed by dialysis. Thereafter, nonpolar compounds were removed by chromatography on a silicic acid column. Acid and nonacid glycosphingolipids were separated by ion change chromatography on a DEAE-cellulose column. In order to separate the nonacid glycosphingolipids from alkali-stable phospholipids, the nonacid fractions were then acetylated and separated on a second silicic acid column, followed by deacetylation and dialysis. Final purifications are performed by chromatography on DEAE-cellulose and silicic acid columns.

After the first characterization by binding assays and LC-ESI/MS, the nonacid glycosphingolipid fractions were separated on Iatrobeads (Iatron Labs) columns eluted with increasing volumes of methanol in chloroform. The fractions obtained were analyzed by thin-layer chromatography and anisaldehyde or resorcinol detection and thereafter pooled according to their mobility on thin-layer chromatograms, resulting in two subfractions from the leiomyosarcoma (denoted fractions Leio-1 and Leio-2) and three subfractions from the liposarcoma (denoted fractions Lipo-1, Lipo-2, and Lipo-3).

### Reference glycosphingolipids

Total acid and nonacid glycosphingolipid fractions were isolated as described (29). Individual glycosphingolipids were isolated by repeated chromatography on silicic acid columns and by HPLC, and identified by mass spectrometry (30, 52) and ^1^H-NMR spectroscopy (53).

### Thin-layer chromatography

Thin-layer chromatography was done on aluminum- or glass-backed silica gel 60 high performance thin-layer chromatography plates (Merck). Glycosphingolipid mixtures (40 μg) or pure glycosphingolipids (2–8 μg) were applied to the plates and eluted with chloroform/methanol/water 60:35:8 (by volume). Chemical detection was done with the anisaldehyde reagent (54) or the resorcinol reagent (55).

### Chromatogram binding assays

The mouse monoclonal antibodies tested for binding to the glycosphingolipids in the chromatogram-binding assay are given in Table 4. Binding of the monoclonal antibodies to glycosphingolipids separated on thin-layer chromatograms was done as described (24). Chromatograms with separated glycosphingolipids were dipped for 1 min in diethylether/*n*-hexane (1:5, by volume) containing 0.5% (w/v) polyisobutylmethacrylate (Sigma-Aldrich). After drying, the chromatograms were soaked in PBS containing 2% bovine serum albumin and 0.1% NaN_3_ (Solution A), for 2 h at room temperature. Suspensions of primary monoclonal antibodies diluted 1:100 – 1:500 (the dilution used for each monoclonal antibody is given in Table 3) in solution A were gently sprinkled over the chromatograms, followed by incubation for 2 h at room temperature, followed by washings with PBS. Then the chromatograms were covered with alkaline phosphate-conjugated goat anti-mouse antibodies (Sigma-Aldrich; A0162) at a dilution of 1:500 and incubated for 1 h. Thereafter, the reactions were visualized with 5-bromo-4-chloro-3-indolyl phosphate/nitro blue tetrazolium chromogenic substrate (Sigma-Aldrich; B5655-25TAB).

**Table 4:** Monoclonal antibodies used in chromatogram binding assays

Antibody	Clone/Designation	Manufacturer/Reference	Dilution	Isotype	Leio	Lipo
Anti-SSEA-4	MC-813–70	eBioScience	1:100	IgG3	+^a^	-
Anti-GD1a	GD1a-1	Millipore	1:100	IgG1	+	+
Anti-NeuAcα3-lactotetra	TR4	Ref. (59)	1:500	IgM	+	-
Anti-NeuAcα3-neolactotetra	LM1:1a	Ref. (60)	1:500	IgM	+	+

a +denotes a binding when 40 μg of the acid glycosphingolipid fraction of the leiomyosarcoma (Leio) or the liposarcoma (Lipo) was applied on the thin-layer chromatogram, while - denotes no binding.

### Endoglycoceramidase digestions and LC-ESI/MS

Endoglycoceramidase II from *Rhodococcus* spp. (Takara Bio Europe S.A.) was used for hydrolysis of the nonacid glycosphingolipids. The glycosphingolipids (50 μg) were resuspended in 100 μl 0.05 M sodium acetate buffer, pH 5.0, containing 120 μg sodium cholate, and sonicated briefly. Thereafter, 1 mU of enzyme was added, and the mixture was incubated at 37 °C for 48 h. The reaction was stopped by the addition of chloroform/methanol/water to the final proportions 8:4:3 (by volume). The oligosaccharide-containing upper phase thus obtained was separated from detergent on a Sep-Pak QMA cartridge (Waters). The eluant containing the oligosaccharides was dried under nitrogen and under nitrogen.

Endoglycoceraminidase I (TCI Europe) (1 μl) was incubated with the acid glycosphingolipids (50 μg) in 5 mM NaAc, pH 5.5, 0.2% Triton-X 100 at 37 °C overnight. The reaction was stopped by the addition of chloroform/methanol/water to the final proportion 8:4:3 (v/v/v). The released oligosaccharides were purified *via* PGC cartridge (Thermo Fisher Scientific). The oligosaccharide-containing 65% acetonitrile elution was dried under nitrogen.

Part of the oligosaccharide samples were reduced by adding 20 μl of 0.5 M NaBH_4_ in 20 mM NaOH to the samples and incubating at 50 °C for overnight. The samples were then acidified by adding 1 μl of glacial acetic acid, and the oligosaccharides were desalted by cation exchange chromatography, and thereafter evaporated to dryness.

Desialylation of oligosaccharides was done by incubation of the oligosaccharides with recombinant AmGH33 A (with α2,3/6-sialic acid specificity) at 37 °C overnight (35). The desialylated oligosaccharides were purified *via* PGC cartridge as described above.

The glycosphingolipid-derived oligosaccharides were resuspended in 50 μl water and analyzed by LC-ESI/MS as described (30). All oligosaccharide-containing samples derived from the glycosphingolipid fractions were analyzed both as native and reduced forms. MS analysis was performed on an LTQ Velos linear ion trap mass spectrometer (Thermo Fisher Scientific). The oligosaccharides were separated on a column (100 × 0.250 mm) packed in-house with 5 μm porous graphite particles (Hypercarb, Thermo-Hypersil). Ultimate 3000 (Thermo Fisher Scientific) with 10 μl loop was used for sample injection at a flow rate of 6 μl/min. The oligosaccharides (3 μl) were injected on to the column and eluted with an acetonitrile gradient (A: 10 mM ammonium bicarbonate; B: 10 mM ammonium bicarbonate in 80% acetonitrile). The gradient (0–45% B) was eluted for 46 min, followed by a wash step with 100% B and equilibration of the column for 24 min. A 30 cm × 50 μm i.d. fused silica capillary was used as transfer line to the ion source.

The oligosaccharides were analyzed in negative ion mode on an LTQ linear quadrupole ion trap mass spectrometer (Thermo Electron). The IonMax standard ESI source on the LTQ mass spectrometer was equipped with a stainless-steel needle kept at −3.5 kV. Compressed air was used as nebulizer gas. The heated capillary was kept at 270 °C, and the capillary voltage was −50 kV. Full-scan (*m/z* 380–2,000, two microscans, maximum 100 ms, target value of 30,000) was performed, followed by data-dependent MS^2^ scans of the three most abundant ions in each scan (2 microscans, maximum 100 ms, target value of 10,000). The threshold for MS^2^ was set to 500 counts. Normalized collision energy was 35%, and an isolation window of 3 u, an activation q = 0.25, and an activation time of 30 ms was used. Data acquisition and processing were conducted with Xcalibur software (Version 2.0.7).

Manual assignment of glycan sequences was done on the basis of knowledge of mammalian biosynthetic pathways, with the assistance of the Glycoworkbench tool (Version 2.1) and by comparison of retention times and MS^2^ spectra of oligosaccharides from reference glycosphingolipids (30). For comparing glycosphingolipid-derived oligosaccharides abundances between samples, individual glycan structure was quantified relative to the total content by integrating the extracted ion chromatogram peak area. The area under the curve of each structure was normalized to the total area under the curve and expressed as a percentage. The peak area was processed by Progenesis QI software (Waters Corporation).

The proposed structures in the formulas in the figures are depicted using the Symbol Nomenclature for Glycomics (SNFG) (56, 57) and nomenclature of fragments defined by Domon and Costello (58).

### Immunohistochemistry

The two leiomyosarcomas (both Trojani grade 2), the two liposarcomas (Trojani grade 1), the normal adipose tissue, and the muscular artery were frozen in liquid nitrogen and kept at −80 °C until use. Cryostat sections were cut at 8 μm and fixed with methanol for 1 min. The sections were blocked with 2.5% horse serum and incubated over night at 5 °C with monoclonal antibodies. The primary antibodies used were anti-Neu5Acα3-lactotetra (TR4 clone; 1:500 dilution in PBS), anti-Neu5Acα3-neolactotetra (LM1:1a clone; 1:500 dilution in PBS), anti-Neu5Acα3-globopenta/SSEA-4 (Abcam; ab16287 [MC813-70]); 1:1000 dilution in PBS), anti-GD1a (Millipore; GD1a-1; 1:500 dilution in PBS), and anti-globopenta/SSEA-3 (Abcam; ab16286 [MC631]); 1:100 dilution in PBS). Peroxidase-conjugated donkey anti-mouse IgG or donkey anti-rat IgG (Jackson Laboratories), respectively, were used as secondary reagents. The reactions were visualized using a Liquid DAB + substrate chromogen system (Agilent), resulting in a brown reaction product. Cell nuclei were counter stained with hematoxylin. Further sections were stained with hematoxylin/eosin to reveal general histology. Images of the slides were obtained by scanning the slides in NanoZoomer-SQ (Hamamatsu). The images were cropped in Affinity designer 2 (2.5.5).

## Data availability

Raw data were uploaded to Glycopost (http://doi.org/10.50821/GLYCOPOST-GPST000483), accessed on Oct 28, 2025).

## Ethical considerations

The study was approved by the Regional Ethics Committee of Gothenburg (No. 2022-00614-01 (decision 2022-03-01)). All patients provided written informed consents before enrollment in the study.

## Supporting information

This article contains supporting information (30, 31, 56, 57, 58).

## Conflict of interest

The authors declare that they have no conflicts of interest with the contents of this article.
