# Supplementary material for: Glycosphingolipids in human abdominal leiomyosarcoma and liposarcoma
Source: J Biol Chem. 2026 Feb 26;302(4):111327. doi: 10.1016/j.jbc.2026.111327 (PMC13022663; doi:10.1016/j.jbc.2026.111327)
Supplement: Supplementary Material [file mmc1.pdf]

Supporting Information

Glycosphingolipids in human abdominal leiomyosarcoma and liposarcoma:

Chunsheng Jin, Anders Thornell, Marta Berndsen, Karin Säljö, Eva Jennische, Susann Teneberg

Supplemental Figure S1. Characterization of the oligosaccharides derived from the total non-acid glycosphingolipids from human leiomyosarcoma and liposarcoma by LC-ESI/MS.

Supplemental Figure S2. Characterization of the oligosaccharides derived from the non-acid glycosphingolipid subfractions from human leiomyosarcoma by LC-ESI/MS.

Supplemental Figure S3. Characterization of the oligosaccharides derived from the non-acid glycosphingolipid subfractions from human liposarcoma by LC-ESI/MS.

Supplemental Figure S4. Characterization of the oligosaccharides derived from the total acid glycosphingolipids from human leiomyosarcoma by LC-ESI/MS.

Supplemental Figure S5. Characterization of the oligosaccharides derived from the complex acid glycosphingolipids from human leiomyosarcoma by mass spectrometry.

Supplemental Fig. S6. Characterization of the oligosaccharides derived from the complex acid glycosphingolipids from human leiomyosarcoma by mass spectrometry.

Supplemental Fig. S7. Immunohistochemistry of Trojani grade 1 liposarcomas, and normal adipose tissue, immunostained with monoclonal antibodies directed against the sialyl-globopenta/SSEA-4 epitope *(A, B* and *E*), and the GD1a ganglioside (*C, D* and *F*).

Fig. S1

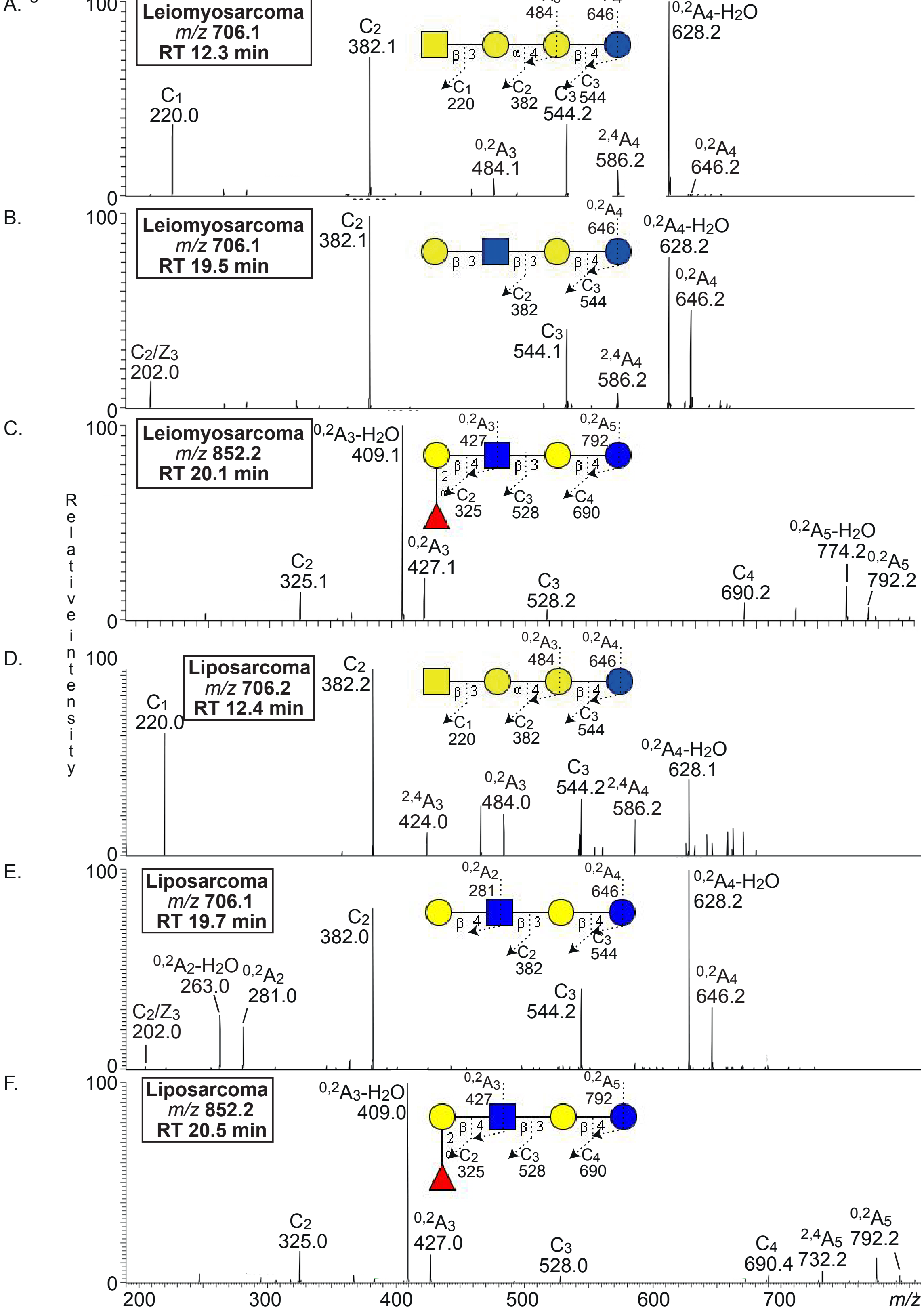
A.
Leiomyosarcoma
m/z 706.1
RT 12.3 min
C1 220.0
C2 382.1
0,2A3 484.1
C3 544.2
2,4A4 586.2
0,2A4-H2O 628.2
0,2A4 646.2
B.
Leiomyosarcoma
m/z 706.1
RT 19.5 min
C2/Z3 202.0
C2 382.1
C3 544.1
2,4A4 586.2
0,2A4-H2O 628.2
0,2A4 646.2
C.
Leiomyosarcoma
m/z 852.2
RT 20.1 min
C2 325.1
0,2A3-H2O 409.1
0,2A3 427.1
C3 528.2
C4 690.2
0,2A5-H2O 774.2
0,2A5 792.2
D.
Liposarcoma
m/z 706.2
RT 12.4 min
C1 220.0
C2 382.2
2,4A3 424.0
0,2A3 484.0
C3 544.2
2,4A4 586.2
0,2A4-H2O 628.1
E.
Liposarcoma
m/z 706.1
RT 19.7 min
C2/Z3 202.0
0,2A2-H2O 263.0
0,2A2 281.0
C2 382.0
C3 544.2
0,2A4-H2O 628.2
0,2A4 646.2
F.
Liposarcoma
m/z 852.2
RT 20.5 min
C2 325.0
0,2A3-H2O 409.0
0,2A3 427.0
C3 528.0
C4 690.4
2,4A5 732.2
0,2A5 792.2
Relative intensity
m/z

Supplemental Figure S1. Characterization of the oligosaccharides derived from the total non-acid glycosphingolipids from human leiomyosarcoma and liposarcoma by LC-ESI/MS. LC-ESI/MS of the oligosaccharides derived from the total non-acid glycosphingolipid fractions from human leiomyosarcoma and liposarcoma by hydrolysis with endoglycoceramidase II from *Rhodococcus* spp. The identification of oligosaccharides was based on their retention times, determined molecular masses, subsequent MS^2 sequencing, and comparison with reference MS^2 spectra.

(*A*) Leiomyosarcoma glycosphingolipid derived oligosaccharides: MS^2 of the $[M-H^+]^-$ ion at *m/z* 706.1 at retention time 12.3 min.

(*B*) Leiomyosarcoma glycosphingolipid derived oligosaccharides: MS^2 of the $[M-H^+]^-$ ion at *m/z* 706.1 at retention time 19.5 min. MS^2 allowed identification of a lacto tetrasaccharide (Galβ3GlcNAcβ3Galβ4Glc). This was concluded from the C-type fragment ions (C_2 at *m/z* 382.1 and C_3 at *m/z* 544.1) identifying a Hex-HexNAc-Hex-Hex sequence, and the D_{1-2} ion at *m/z* 202.0, which is obtained by a C_2/Z_2 double cleavage, and diagnostic for a 3-substituted HexNAc (1).

(*C*) Leiomyosarcoma glycosphingolipid derived oligosaccharides: MS^2 of the $[M-H^+]^-$ ion at *m/z* 852.2 at retention time 20.1 min.

(*D*) Liposarcoma glycosphingolipid derived oligosaccharides: MS^2 of the $[M-H^+]^-$ ion at *m/z* 706.1 at retention time 12.4 min.

(*E*) Liposarcoma glycosphingolipid derived oligosaccharides: The MS^2 spectrum of the $[M-H^+]^-$ ion at *m/z* 706.2 at retention time 18.8 min demonstrated two co-eluting compounds, a lacto tetrasaccharide (Galβ3GlcNAcβ3Galβ4Glc) and a neolacto tetrasaccharide (Galβ4GlcNAcβ3Galβ4Glc). There was a C_2 ion at *m/z* 382.0 and a C_3 ion at *m/z* 544.2 demonstrating a Hex-HexNAc-Hex-Hex sequence. There were also two diagnostic ions. Firstly, a D_{1-2} ion at *m/z* 202.0, which is obtained by a C_2/Z_2 double cleavage, and diagnostic for a 3-substituted HexNAc (1), *i.e.* a type 1 core chain, and secondly a $^{0,2}A_2$ fragment ion at *m/z* 281.0 which demonstrated a terminal Hex-HexNAc sequence with a 4-substituted HexNAc (1, 2), *i.e.* a type 2 core chain.

(*F*) Liposarcoma glycosphingolipid derived oligosaccharides: MS^2 of the $[M-H^+]^-$ ion at *m/z* 852.2 at retention time 20.5 min.

The MS^2 spectra of the $[M-H^+]^-$ ion at *m/z* 706.1 at retention time 12.3-12.4 min (*A* and *D*) identified a globo tetrasaccharide (GalNAcβ3Galα4Galβ4Glc) by the C-type fragment ion series (C_1 at *m/z* 220.0, C_2 at *m/z* 382.1, and C_3 at *m/z* 544.2), demonstrating a HexNAc-Hex-Hex-Hex sequence. The $^{0,2}A_3$ fragment ion at *m/z* 484.0/1 demonstrated a 4-substituted Hex (1, 2).

The MS^2 spectra of the $[M-H^+]^-$ ion at *m/z* 852.2 eluting at 20.1-20.5 min (*C* and *F*) had series of C type fragment ions (C_2 at *m/z* 325.0/1, C_3 at *m/z* 528.0/2, and C_4 at *m/z* 690.2/4), identifying a pentasaccharide with Fuc-Hex-HexNAc-Hex-Hex sequence. The $^{0,2}A_3$ fragment ion at *m/z* 427.0/1 is characteristic for 4-substituted HexNAc, *i.e.* a type 2 carbohydrate chain (1, 2). This demonstrated an H type 2 pentasaccharide (Fucα2Galβ4GlcNAcβ3Galβ4Glc).

The proposed structures in the interpretation formulas are depicted using the Symbol Nomenclature for Glycomics (SNFG) (3, 4), and nomenclature of fragments defined by Domon and Costello (5).

Fig. S2

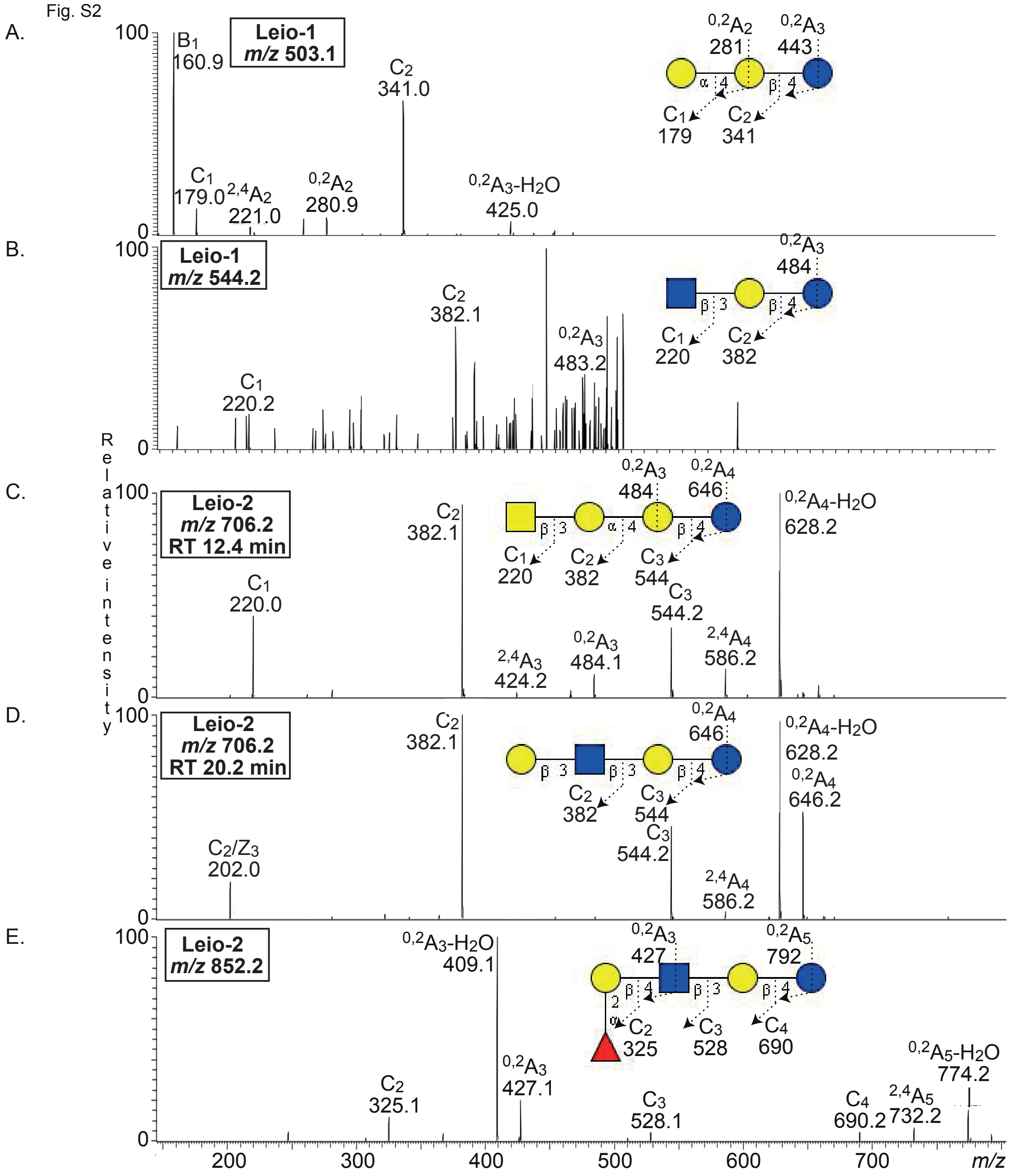

A.
Leio-1
m/z 503.1
B1
160.9
C1
179.0
2,4A2
221.0
0,2A2
280.9
C2
341.0
0,2A3-H2O
425.0
0,2A2
281
0,2A3
443
C1
179
C2
341
B.
Leio-1
m/z 544.2
C1
220.2
C2
382.1
0,2A3
483.2
0,2A3
484
C1
220
C2
382
C.
Leio-2
m/z 706.2
RT 12.4 min
C1
220.0
C2
382.1
2,4A3
424.2
0,2A3
484.1
C3
544.2
2,4A4
586.2
0,2A4-H2O
628.2
0,2A3
484
0,2A4
646
C1
220
C2
382
C3
544
D.
Leio-2
m/z 706.2
RT 20.2 min
C2/Z3
202.0
C2
382.1
C3
544.2
2,4A4
586.2
0,2A4-H2O
628.2
0,2A4
646.2
0,2A4
646
C2
382
C3
544
E.
Leio-2
m/z 852.2
C2
325.1
0,2A3-H2O
409.1
0,2A3
427.1
C3
528.1
C4
690.2
2,4A5
732.2
0,2A5-H2O
774.2
0,2A3
427
0,2A5
792
C2
325
C3
528
C4
690
Relative intensity
m/z

Supplemental Figure S2. Characterization of the oligosaccharides derived from the non-acid glycosphingolipid subfractions from human leiomyosarcoma by LC-ESI/MS. LC-ESI/MS of the oligosaccharides derived from the non-acid glycosphingolipid subfractions Leio-1 and Leio-2 from human leiomyosarcoma by hydrolysis with endoglycoceramidase II from *Rhodococcus* spp. The identification of oligosaccharides was based on their retention times, determined molecular masses, subsequent MS^2 sequencing, and comparison with reference MS^2 spectra.

(*A*) Leiomyosarcoma glycosphingolipid derived oligosaccharides in fraction Leio-1: MS^2 of the $[M-H^+]^-$ ion at *m/z* 503.1 at retention time 10.4 min gave a C-type fragment ions series (C_1 at *m/z* 179.0 and C_2 at *m/z* 341.0) identifying a Hex-Hex-Hex sequence. The $^{2,4}A_2$ fragment ion at *m/z* 221.0 and $^{0,2}A_2$ fragment ion at *m/z* 280.9 demonstrated that the penultimate Hex was 4-substituted (1, 2). Taken together this identified a globo trisaccharide (Galα4Galβ4Glc).

(*B*) Leiomyosarcoma glycosphingolipid derived oligosaccharides in fraction Leio-1: MS^2 of the $[M-H^+]^-$ ion at *m/z* 544.2 at retention time 15.4 min gave two C-type fragment ions (C_1 at *m/z* 220.2 and C_2 at *m/z* 382.1) identifying a HexNAc-Hex-Hex sequence. There was no $^{0,2}A_2$ fragment ion at *m/z* 322, as in the ganglio trisaccharide. Thus, a lacto trisaccharide (GlcNAcβ3Galβ4Glc) was identified.

(*C*) Leiomyosarcoma glycosphingolipid derived oligosaccharides in fraction Leio-2: MS^2 of the $[M-H^+]^-$ ion at *m/z* 706.2 at retention time 12.4 min identified a globo tetrasaccharide (GalNAcβ3Galα4Galβ4Glc) by the C-type fragment ion series (C_1 at *m/z* 220.0, C_2 at *m/z* 382.1, and C_3 at *m/z* 544.2), demonstrating a HexNAc-Hex-Hex-Hex sequence and the $^{0,2}A_3$ fragment ion at *m/z* 484.1 demonstrating a 4-substituted Hex (1, 2).

(*D*) Leiomyosarcoma glycosphingolipid derived oligosaccharides in fraction Leio-2: MS^2 of the $[M-H^+]^-$ ion at *m/z* 706.2 at retention time 20.2 min allowed identification of a lacto tetrasaccharide (Galβ3GlcNAcβ3Galβ4Glc). This was concluded from the C-type fragment ions (C_2 at *m/z* 382.1 and C_3 at *m/z* 544.2) identifying a Hex-HexNAc-Hex-Hex sequence, and the D_{1-2} ion at *m/z* 202.0, which is obtained by a C_2/Z_2 double cleavage, and diagnostic for a 3-substituted HexNAc (1).

(*E*) Leiomyosarcoma glycosphingolipid derived oligosaccharides in fraction Leio-2: MS^2 of the $[M-H^+]^-$ ion at *m/z* 852.2 at retention time 21.0 min demonstrated an H type 2 pentasaccharide (Fucα2Galβ4GlcNAcβ3Galβ4Glc). The MS^2 spectrum had a series of C type fragment ions (C_2 at *m/z* 325.1, C_3 at *m/z* 528.1, and C_4 at *m/z* 690.2), identifying a pentasaccharide with Fuc-Hex-HexNAc-Hex-Hex sequence. The $^{0,2}A_3$ fragment ion at *m/z* 427.1 is characteristic for 4-substituted HexNAc, *i.e.* a type 2 carbohydrate chain (1, 2).

The proposed structures in the interpretation formulas are depicted using the Symbol Nomenclature for Glycomics (SNFG) (3, 4), and nomenclature of fragments defined by Domon and Costello (5).

Fig. S3

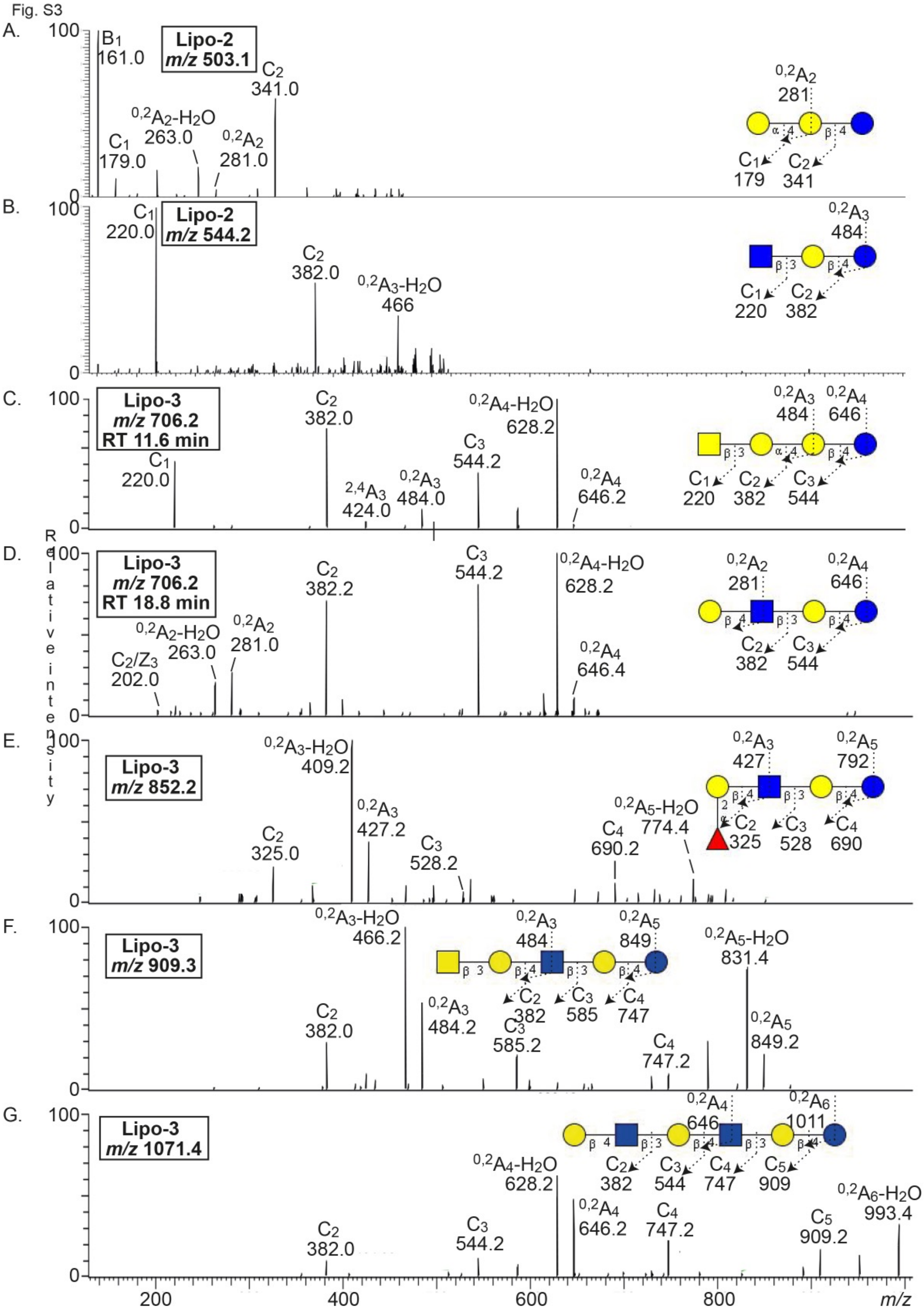
A.
B.
C.
D.
E.
F.
G.
Relative intensity
Lipo-2 m/z 503.1
B1 161.0
C1 179.0
0,2A2-H2O 263.0
0,2A2 281.0
C2 341.0
Lipo-2 m/z 544.2
C1 220.0
C2 382.0
0,2A3-H2O 466
Lipo-3 m/z 706.2 RT 11.6 min
C1 220.0
C2 382.0
2,4A3 424.0
0,2A3 484.0
C3 544.2
0,2A4-H2O 628.2
0,2A4 646.2
Lipo-3 m/z 706.2 RT 18.8 min
C2/Z3 202.0
0,2A2-H2O 263.0
0,2A2 281.0
C2 382.2
C3 544.2
0,2A4-H2O 628.2
0,2A4 646.4
Lipo-3 m/z 852.2
C2 325.0
0,2A3-H2O 409.2
0,2A3 427.2
C3 528.2
C4 690.2
0,2A5-H2O 774.4
Lipo-3 m/z 909.3
C2 382.0
0,2A3-H2O 466.2
0,2A3 484.2
C3 585.2
C4 747.2
0,2A5-H2O 831.4
0,2A5 849.2
Lipo-3 m/z 1071.4
C2 382.0
C3 544.2
0,2A4-H2O 628.2
0,2A4 646.2
C4 747.2
C5 909.2
0,2A6-H2O 993.4
m/z

Supplemental Figure S3. Characterization of the oligosaccharides derived from the non-acid glycosphingolipid subfractions from human liposarcoma by LC-ESI/MS. LC-ESI/MS of the oligosaccharides derived from the non-acid glycosphingolipid subfractions Lipo-2 and Lipo-2 from human liposarcoma by hydrolysis with endoglycoceramidase II from *Rhodococcus* spp. The identification of oligosaccharides was based on their retention times, determined molecular masses, subsequent MS^2 sequencing, and comparison with reference MS^2 spectra.

(*A*) Liposarcoma glycosphingolipid derived oligosaccharides in fraction Lipo-2: A C-type fragment ions series (C_1 at *m/z* 179.0 and C_2 at *m/z* 341.0) identifying a Hex-Hex-Hex sequence was present in the MS^2 spectrum of the $[M-H^+]^-$ ion at *m/z* 503.1 at retention time 11.4 min. The $^{0,2}A_2$ fragment ion at *m/z* 281.0 demonstrated that the penultimate Hex was 4-substituted (1, 2). Thus, a globo trisaccharide (Galα4Galβ4Glc) was identified.

(*B*) Liposarcoma glycosphingolipid derived oligosaccharides in fraction Lipo-2: The MS^2 spectrum obtained of the $[M-H^+]^-$ ion at *m/z* 544.2 at retention time 16.4 min had two C-type fragment ions (C_1 at *m/z* 220.0 and C_2 at *m/z* 382.0) identifying a HexNAc-Hex-Hex sequence. There was no $^{0,2}A_2$ fragment ion at *m/z* 322, as in the ganglio trisaccharide. Taken together this identified a lacto trisaccharide (GlcNAcβ3Galβ4Glc).

(*C*) Liposarcoma glycosphingolipid derived oligosaccharides in fraction Lipo-3: A globo tetrasaccharide (GalNAcβ3Galα4Galβ4Glc) was identified by MS^2 of the $[M-H^+]^-$ ion at *m/z* 706.2 at retention time 11.4 min. This was concluded from the C-type fragment ion series (C_1 at *m/z* 220.0, C_2 at *m/z* 382.0, and C_3 at *m/z* 544.2), demonstrating a HexNAc-Hex-Hex-Hex sequence, along with the $^{0,2}A_3$ fragment ion at *m/z* 484.2, which demonstrated a 4-substituted Hex (1, 2).

(*D*) Liposarcoma glycosphingolipid derived oligosaccharides in fraction Leio-3: The MS^2 spectrum of the $[M-H^+]^-$ ion at *m/z* 706.2 at retention time 18.8 min had a C_2 ion at *m/z* 382.0 and the C_3 ion at *m/z* 544.2 demonstrating a Hex-HexNAc-Hex-Hex sequence. There were two diagnostic ions. Firstly, a D_{1-2} ion at *m/z* 202.0, which is obtained by a C_2/Z_2 double cleavage, and diagnostic for a 3-substituted HexNAc (1),), *i.e.* a type 1 core chain, and secondly a $^{0,2}A_2$ fragment ion at *m/z* 281.0 which demonstrated a terminal Hex-HexNAc sequence with a 4-substituted HexNAc (1, 2), *i.e.* a type 2 core chain. Thus there were two co-eluting compounds, a lacto tetrasaccharide (Galβ3GlcNAcβ3Galβ4Glc) and a neolacto tetrasaccharide (Galβ4GlcNAcβ3Galβ4Glc).

(*E*) Liposarcoma glycosphingolipid derived oligosaccharides in fraction Lipo-3: MS^2 of the $[M-H^+]^-$ ion at *m/z* 852.2 at retention time 20.6 min demonstrated an H type 2 pentasaccharide (Fucα2Galβ4GlcNAcβ3Galβ4Glc). This conclusion was based on the series of C type fragment ions (C_2 at *m/z* 325.1, C_3 at *m/z* 528.2 and C_4 at *m/z* 690.2), which identified a pentasaccharide with Fuc-Hex-HexNAc-Hex-Hex sequence, and the $^{0,2}A_3$ fragment ion at *m/z* 427.1, which demonstrated a 4-substituted HexNAc, *i.e.* a type 2 carbohydrate chain (1, 2).

(*F*) Liposarcoma glycosphingolipid derived oligosaccharides in fraction Lipo-3: MS^2 of the molecular ion at *m/z* 909.3 at retention time 33.9 min gave a series of C-type fragment ions (C_2 at *m/z* 382.0, C_3 at *m/z* 585.2 and C_4 at *m/z* 747.2) demonstrating a HexNAc-Hex-HexNAc-Hex-Hex sequence. A type 2 core chain was identified by the $^{0,2}A_4$ ion at *m/z* 484.2. Thus an x_2 pentasaccharide (GalNAcβ3Galβ4GlcNAcβ3Galβ4Glc) was identified.

(*G*) Liposarcoma glycosphingolipid derived oligosaccharides in fraction Lipo-3: A neolacto hexasaccharide (Galβ4GlcNAcβ3Galβ4GlcNAcβ3Galβ4Glc) was characterized by MS^2 of the $[M-H^+]^-$ ion at *m/z* 1071.4 eluting at 24.7 min. This was deduced from the C-type fragment ion series (C_2 at *m/z* 382.0, C_3 at *m/z* 544.2, C_4 at *m/z* 747.2, and C_5 at *m/z* 909.4), demonstrating a Hex-HexNAc-Hex-HexNAc-Hex-Hex carbohydrate sequence, along with the $^{0,2}A_4$ fragment ion at *m/z* 646.2, which demonstrated 4-substitution of the innermost HexNAc (1, 2). The structures in the interpretation formulas are depicted using the Symbol Nomenclature for Glycomics (SNFG) (3, 4), and nomenclature of fragments defined by Domon and Costello (5).

Fig. S4

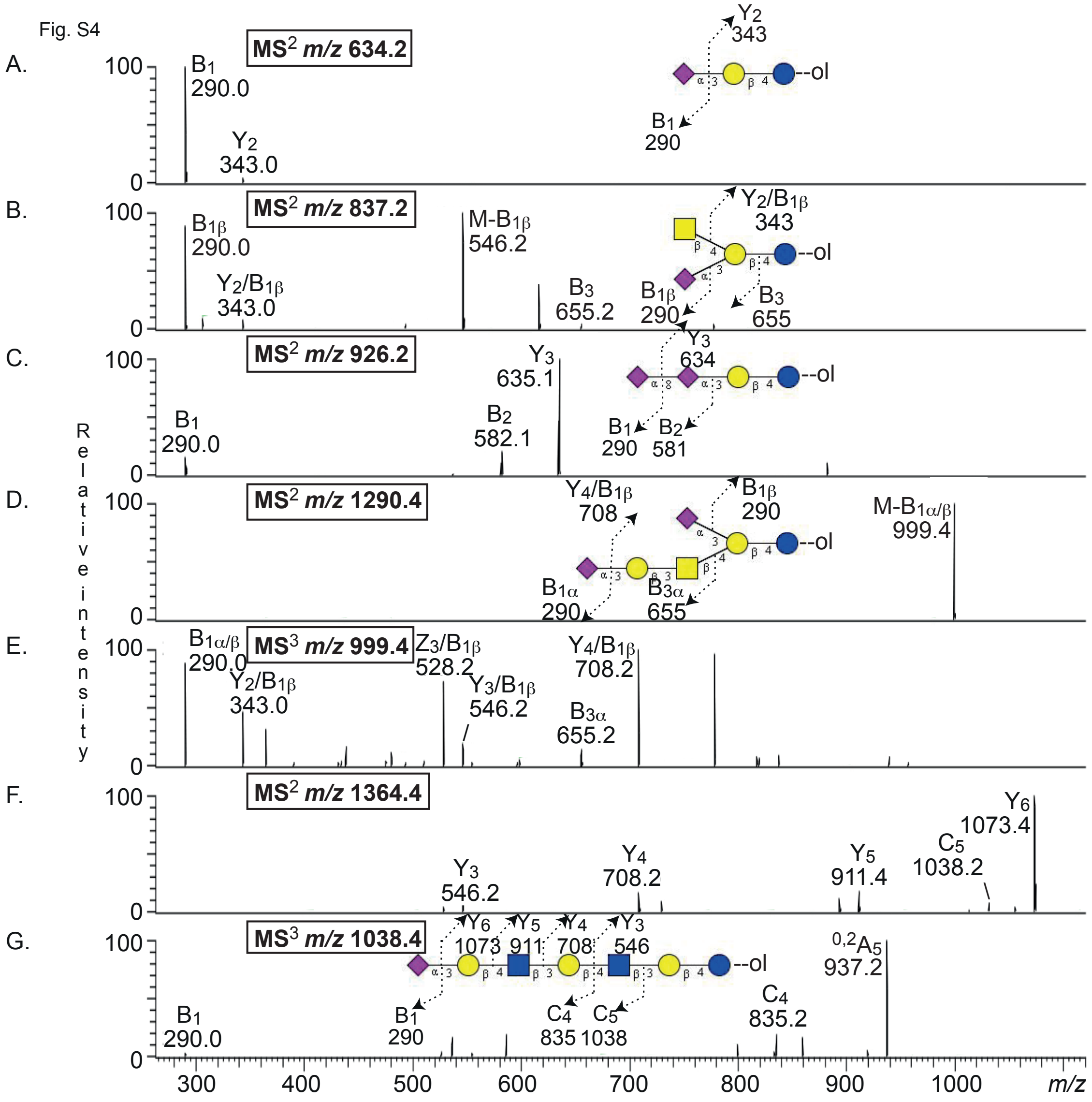
A.
B.
C.
D.
E.
F.
G.
Relative intensity
MS2 m/z 634.2
MS2 m/z 837.2
MS2 m/z 926.2
MS2 m/z 1290.4
MS3 m/z 999.4
MS2 m/z 1364.4
MS3 m/z 1038.4
m/z
--ol

Supplemental Figure S4. Characterization of the oligosaccharides derived from the total acid glycosphingolipids from human leiomyosarcoma by LC-ESI/MS. LC-ESI/MS of the reduced oligosaccharides derived from the total acid glycosphingolipid fraction from human leiomyosarcoma by hydrolysis with endoglycoceramidase I. The identification of oligosaccharides was based on their retention times, determined molecular masses, subsequent MS^2 sequencing, and comparison with reference MS^2 spectra.

(*A*) Reduced acid leiomyosarcoma oligosaccharides: MS^2 of the $[M-H^+]^-$ ion at *m/z* 634.2 at retention time 15.6 min gave a Y_2 ion at *m/z* 343.0 and a B_1 ion at *m/z* 290.0 demonstrating an oliogosaccharide with Neu5Ac-Hex-Hex sequence *i.e.* the GM3 trisaccharide (Neu5Acα3Galβ4Glc).

(*B*) Reduced acid leiomyosarcoma oligosaccharides: MS^2 of the $[M-H^+]^-$ ion at *m/z* 837.2 at retention time 15.4 min. The MS^2 spectrum had a $B_{1\beta}$ ion at *m/z* 290.0, and a $Y_2/B_{1\beta}$ ion at *m/z* 343.0. Taken together with the B type ion at *m/z* 655 (B_3) this identified an oligosaccharide with HexNAc(Neu5Ac)Hex-Hex sequence, most likely the GM2 tetrasaccharide (GalNAcβ4(Neu5Acα3)Galβ4Glc).

(*C*) Reduced acid leiomyosarcoma oligosaccharides: MS^2 of the $[M-H^+]^-$ ion at *m/z* 926.2 at retention time 13.4 min. A terminal Neu5Ac-Neu5Ac sequence was indicated by the B ions (B_1 at *m/z* 290.0 and B_2 at *m/z* 582.1) In addition, there was a Y_3 ion at *m/z* 635.1. Taken together this indicated an oliogosaccharide with Neu5Ac-Neu5Ac-Hex-Hex sequence, as the NeuAc-GD3 tetrasaccharide (Neu5Acα8Neu5Acα3Galβ4Glc).

(*D*) Reduced acid leiomyosarcoma oligosaccharides: MS^2 of the $[M-H^+]^-$ ion at *m/z* 1290.4 at retention time 20.4 min, along with MS^3 ion the ion at *m/z* 999.4 in (*E*), gave a number of B, Z and Y type ions identifying an oliogosaccharide with Neu5Ac-Hex-HexNAc(Neu5Ac)Hex-Hex sequence, *i.e.* the GD1a hexasaccharide (Neu5Acα3Galβ3GalNAcβ4-(Neu5Acα3)Galβ4Glc).

(*E*) MS^3 of the ion at *m/z* 999.4 in (*D*).

(*F*) Reduced acid leiomyosarcoma oligosaccharides: MS^2 of the $[M-H^+]^-$ ion at *m/z* 1364.4 at retention time 25.5 min, and MS^3 of the ion at *m/z* 1038.4 in (*G*), gave a B_1 ion at *m/z* 290.0 identifying a terminal Neu5Ac, and a number of C type ions (C_4 at *m/z* 835.2 and C_5 at *m/z* 1038.2), which along with the series of Y ions (Y_3 at *m/z* 546.2, Y_4 at *m/z* 708.2, Y_5 at *m/z* 911.4, and Y_6 at *m/z* 1073.4) demonstrated an oligosaccharide with Neu5Ac-Hex-HexNAc-Hex-HexNAc-Hex-Hex sequence. The $^{0,2}A_5$ ion at *m/z* 937.2 showed that the innermost HexNAc was substituted at C-4 (1, 2). Taken together this demonstrated a Neu5Ac-neolacto heptasaccharide (Neu5Acα3Galβ4GlcNAcβ3Galβ4GlcNAcβ3Galβ4Glc).

(*G*) MS^3 of the ion at *m/z* 1038.4 in (*F*).

The proposed structures in the interpretation formulas are depicted using the Symbol Nomenclature for Glycomics (SNFG) (3, 4), and nomenclature of fragments defined by Domon and Costello (5).

Fig. S5

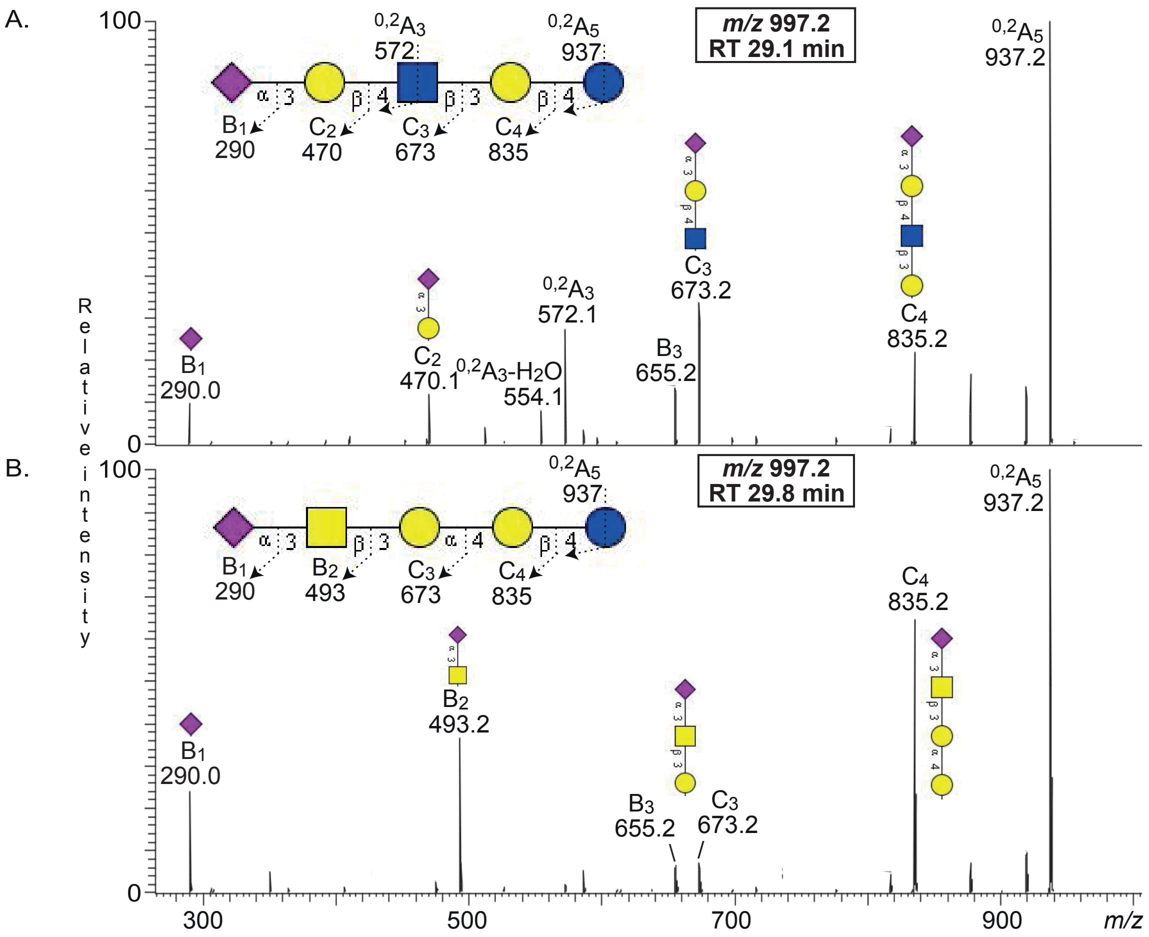

Supplemental Figure S5. Characterization of the oligosaccharides derived from the complex acid glycosphingolipids from human leiomyosarcoma by mass spectrometry. LC-ESI/MS of the oligosaccharides derived from the acid fraction from human leiomyosarcoma by hydrolysis with endoglycoceramidase I. The identification of oligosaccharides was based on their retention times, determined molecular masses, and subsequent MS^2 sequencing.

(*A*) MS^2 of the $[M\text{-}H^+]^-$ ion at *m/z* 997.2 at retention time 29.1 min. The series of B and C fragment ions (B_1 at *m/z* 290.0, C_2 at *m/z* 470.1, B_3 at *m/z* 655.2, C_3 at *m/z* 673.2, and C_4 at *m/z* 835.2) demonstrated a Neu5Ac-Hex-HexNAc-Hex-Hex sequence. The $^{0,2}A_3$ ion at *m/z* 572.1 showed that the HexNAc was substituted at C-4. Thus a sialyl-neolactotetra saccharide (Neu5Acα3/α6Galβ4GlcNAcβ3Galβ4Glc) was identified.

(*B*) MS^2 of the $[M\text{-}H^+]^-$ ion at *m/z* 997.2 at retention time 29.8 min. The B and C ion series (B_1 at *m/z* 290.0, B_2 at *m/z* 493.2, B_3 at *m/z* 655.2, C_3 at *m/z* 673.2, and C_4 at *m/z* 835.2) identified a Neu5Ac-HexNAc-Hex-Hex-Hex sequence, *i.e.* a sialyl-globotetra saccharide (Neu5Acα3GalNAcβ3Galα4Galβ4Glc).

The proposed structures in the interpretation formulas are depicted using the Symbol Nomenclature for Glycomics (SNFG) (3, 4), and nomenclature of fragments defined by Domon and Costello (5).

Fig. S6

De-sialylated acid oligosaccharides

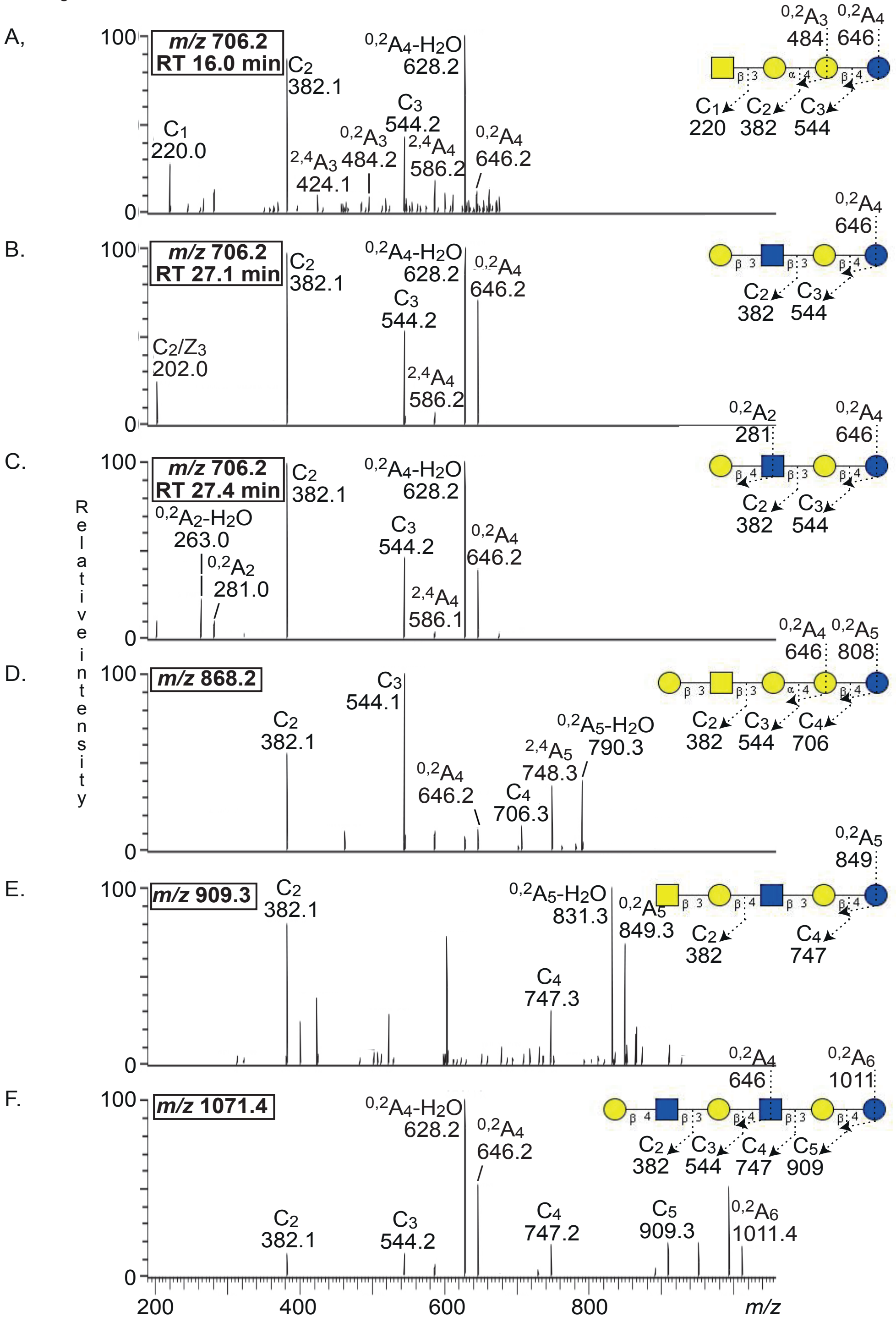

Supplemental Fig. S6. Characterization of the oligosaccharides derived from the complex acid glycosphingolipids from human leiomyosarcoma by mass spectrometry. LC-ESI/MS of the the de-sialylated oligosaccharides derived from the acid fraction from human leiomyosarcoma by hydrolysis with endoglycoceramidase I. The identification of oligosaccharides was based on their retention times, determined molecular masses, and subsequent MS^2 sequencing.

(*A*) MS^2 of the $[M\text{-}H^+]^-$ ion at *m/z* 706.2 at retention time 16.0 min. MS^2 identified a globo tetrasaccharide (GalNAcβ3Galα4Galβ4Glc) by the C-type fragment ion series (C_1 at *m/z* 220.0, C_2 at *m/z* 382.1, and C_3 at *m/z* 544.2), demonstrating a HexNAc-Hex-Hex-Hex sequence and the $^{0,2}A_3$ fragment ion at *m/z* 484.2 demonstrating a 4-substituted Hex (1, 2).

(*B*) MS^2 of the $[M\text{-}H^+]^-$ ion at *m/z* 706.2 at retention time 27.1 min. MS^2 gave identification of a lacto tetrasaccharide (Galβ3GlcNAcβ3Galβ4Glc). This was concluded from the C-type fragment ions (C_2 at *m/z* 382.1 and C_3 at *m/z* 544.2) identifying a Hex-HexNAc-Hex-Hex sequence, and the $D_{1\text{-}2}$ ion at *m/z* 202.0, which is obtained by a C_2/Z_2 double cleavage, and diagnostic for a 3-substituted HexNAc (*1*).

(*C*) MS^2 of the $[M\text{-}H^+]^-$ ion at *m/z* 706.2 at retention time 27.4 min. The MS^2 spectrum had a $^{0,2}A_2$ fragment ion at *m/z* 281.0 demonstrating a terminal Hex-HexNAc sequence with a 4-substituted HexNAc, *i.e.* a type 2 core chain (Galβ4GlcNAc). In combination with the C_2 ion at *m/z* 382.1 and the C_3 ion at *m/z* 544.2, this demonstrated a neolacto tetrasaccharide (Galβ4GlcNAcβ3Galβ4Glc).

(*D*) MS^2 of the $[M\text{-}H^+]^-$ ion at *m/z* 868.2 at retention time 19.0 min. The MS^2 spectrum had a C ions series (C_2 at *m/z* 382.1, C_3 at *m/z* 544.1, and C_4 at *m/z* 706.3), which demonstrated a Hex-HexNAc-Hex-Hex-Hex oligosaccharide. The $^{0,2}A_4$ fragment ion at *m/z* 646.2 demonstrated 4-substitution of the internal Hex. Thus, a globo/SSEA-3 pentasaccharide (Galβ3GalNAcβ3Galα4Galβ4Glc) was identified.

(*E*) MS^2 of the $[M\text{-}H^+]^-$ ion at *m/z* 909.3 at retention time 26.6 min. MS^2 gave a number of C-type fragment ions (C_2 at *m/z* 382.1 and C_4 at *m/z* 747.2) demonstrating a HexNAc-Hex-HexNAc-Hex-Hex sequence like the x_2 pentasaccharide (GalNAcβ3Galβ4GlcNAcβ3-Galβ4Glc).

(*F*) MS^2 of the $[M\text{-}H^+]^-$ ion at *m/z* 1071.4 at retention time 36.2 min. A neolacto hexasaccharide (Galβ4GlcNAcβ3Galβ4GlcNAcβ3Galβ4Glc) was characterized by MS^2. This was deduced from the C-type fragment ion series (C_2 at *m/z* 382.1, C_3 at *m/z* 544.2, C_4 at *m/z* 747.2, and C_5 at *m/z* 909.3), demonstrating a Hex-HexNAc-Hex-HexNAc-Hex-Hex carbohydrate sequence, along with the $^{0,2}A_4$ fragment ion at *m/z* 646.2, which demonstrated 4-substitution of the innermost HexNAc.

The proposed structures in the interpretation formulas are depicted using the Symbol Nomenclature for Glycomics (SNFG) (3, 4), and nomenclature of fragments defined by Domon and Costello (5).

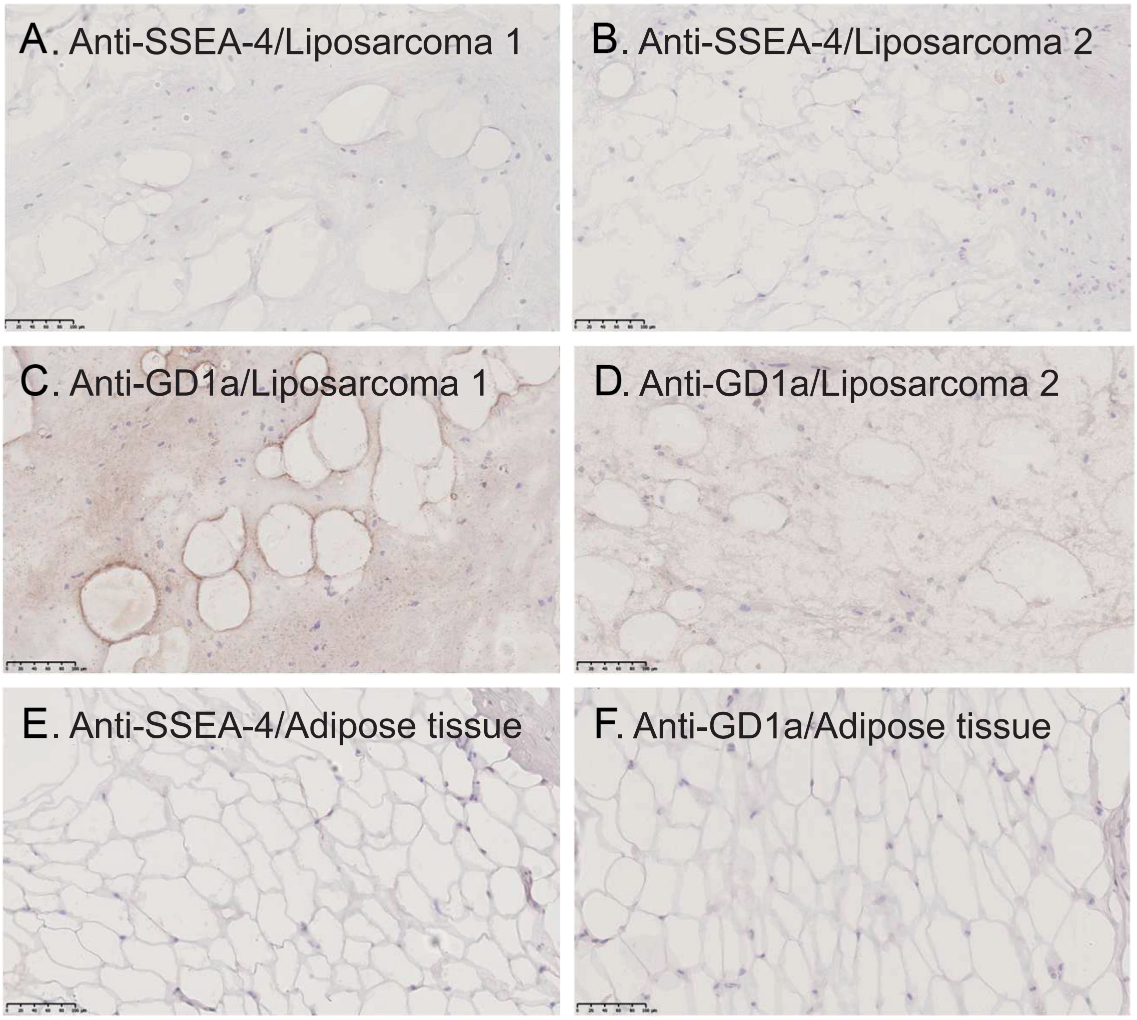

Supplemental Fig. S7. Immunohistochemistry of Trojani grade 1 liposarcomas, and normal adipose tissue, immunostained with monoclonal antibodies directed against the sialyl-globopenta/SSEA-4 epitope *(A, B* and *E*), and the GD1a ganglioside (*C, D* and *F*). Antibody binding was demonstrated with a peroxidase-conjugated secondary antibody/DAB giving a brown reaction product. Nuclei were counterstained with hematoxylin, blue. Magnification x 40. Bar 100 µm.

Liposarcoma 1 (*A*) and liposarcoma 2 (*B*) were completely unstained by the anti-sialyl-globopenta/SSEA-4 antibody. In liposarcoma 1 (*C*) the anti-GD1a antibody bound to materials surrounding the adipocytes, but there was no binding to the small atypical cells. There was no distinct binding of the anti-GD1a antibody to liposarcoma 2 (*D*). There was no staining of the normal adipose tissue by the anti-sialyl-globopenta/SSEA-4 antibody or the anti-GD1a antibody (*E* and *F*).

REFERENCES

1. Chai, W., Piskarev, V., Lawson, A. M. (2001) Negative-ion electrospray mass spectrometry of neutral underivatized oligosaccharides. *Anal. Chem*. **73,** 651-657
2. Karlsson, H. Halim, A., Teneberg, S. (2010) Differentiation of glycosphingolipid-derived glycan structural isomers by liquid chromatography-mass spectrometry. *Glycobiology* **20,** 1103-1116
3. Varki, A., Cummings, R. D., Aebi, M., Packer, N. H., Seeberger, P. H., Esko, J. D., *et al*. (2015) Symbol nomenclature for graphical representations of glycans. *Glycobiology* **25,** 1323–1324
4. Neelamegham, S., Aoki-Kinoshita, K., Bolton, E., Frank, M., Lisacek, F., Lütteke, T., *et al*. The SNFG Discussion Group. (2019) Updates to the symbol nomenclature for glycans guidelines. *Glycobiology* **29,** 620-624
5. Domon, B., Costello, C. (1988) A systematic nomenclature for carbohydrate fragmentations in FAB-MS/MS spectra of glycoconjugates. *Glycoconj. J.* **5,** 397-409
